# Paralogous Radiations of PIN Proteins with Multiple Origins of Noncanonical PIN Structure

**DOI:** 10.1093/molbev/msu147

**Published:** 2014-04-23

**Authors:** Tom Bennett, Samuel F. Brockington, Carl Rothfels, Sean W. Graham, Dennis Stevenson, Toni Kutchan, Megan Rolf, Philip Thomas, Gane Ka-Shu Wong, Ottoline Leyser, Beverley J. Glover, C. Jill Harrison

**Affiliations:** ^1^Department of Plant Sciences, University of Cambridge, Cambridge, United Kingdom; ^2^Sainsbury Laboratory, University of Cambridge, Cambridge, United Kingdom; ^3^Department of Zoology, University of British Columbia, Vancouver, British Colombia, Canada; ^4^UBC Botanical Garden Campbell Building, Vancouver, British Colombia, Canada; ^5^Molecular Systematics, The New York Botanical Garden, Bronx, NY; ^6^Danforth Center, St. Louis, MO; ^7^Royal Botanic Gardens Edinburgh, 20A Inverleith Row, Edinburgh, United Kingdom; ^8^Department of Medicine, University of Alberta, Edmonton, Alberta, Canada; ^9^Department of Biological Sciences, University of Alberta, Edmonton, Alberta, Canada; ^10^BGI-Shenzhen, Beishan Industrial Zone, Yantian District, Shenzhen, China

**Keywords:** auxin, auxin transport, PIN protein, plant evolution, phylogeny, protein structure

## Abstract

The plant hormone auxin is a conserved regulator of development which has been implicated in the generation of morphological novelty. PIN-FORMED1 (PIN) auxin efflux carriers are central to auxin function by regulating its distribution. PIN family members have divergent structures and cellular localizations, but the origin and evolutionary significance of this variation is unresolved. To characterize PIN family evolution, we have undertaken phylogenetic and structural analyses with a massive increase in taxon sampling over previous studies. Our phylogeny shows that following the divergence of the bryophyte and lycophyte lineages, two deep duplication events gave rise to three distinct lineages of PIN proteins in euphyllophytes. Subsequent independent radiations within each of these lineages were taxonomically asymmetric, giving rise to at least 21 clades of PIN proteins, of which 15 are revealed here for the first time. Although most PIN protein clades share a conserved canonical structure with a modular central loop domain, a small number of noncanonical clades dispersed across the phylogeny have highly divergent protein structure. We propose that PIN proteins underwent sub- and neofunctionalization with substantial modification to protein structure throughout plant evolution. Our results have important implications for plant evolution as they suggest that structurally divergent PIN proteins that arose in paralogous radiations contributed to the convergent evolution of organ systems in different land plant lineages.

## Introduction

The earliest land plants are thought to have resembled modern bryophytes that have a haploid-dominant life cycle and a single diploid stem (Graham et al. 2000; Gensel 2008; Langdale and Harrison 2008; Harrison et al. 2010), and the divergence of the vascular plants from their bryophyte sisters was underpinned by a suite of developmental and architectural innovations (Langdale and Harrison 2008). Although several characteristics have a monophyletic origin (e.g., diploid dominance, indeterminacy, branching, and tracheids), leaves and roots evolved independently in lycophytes, ferns, and seed plants (Langdale and Harrison 2008). Similarly, indeterminate branching shoots with leaves and rooting functions evolved by convergence in the haploid shoot systems of mosses and liverworts (Qiu et al. 2006; Menand et al. 2007; Langdale and Harrison 2008). The mechanisms underlying morphological convergence in plant evolution are largely unknown, although a key contribution of the plant hormone auxin has recently been postulated (Finet and Jaillais 2012).

Auxin (indole-3-acetic acid, IAA) regulates many aspects of land plant development via a conserved signaling pathway in which transcriptional responses are finely tuned in response to auxin levels (Lau et al. 2009; Prigge et al. 2010; Calderón Villalobos et al. 2012). Spatial specificity of response is generated by the regulated distribution of auxin; synthesis, degradation, conjugation, and transport all contribute (reviewed in Woodward and Bartel [2005]), with transport playing a pivotal role. In angiosperms, long-range polar auxin transport co-ordinates shoot to root signaling and determines branching patterns (Bhalerao et al. 2002; Bennett et al. 2006). Short range auxin transport modulates meristem activity (Reinhardt et al. 2003; Blilou et al. 2005), leaf initiation patterns (Reinhardt et al. 2003; Heisler et al. 2005), vascular patterning and differentiation (Scarpella et al. 2006), directional growth responses (Friml et al. 2002; Moulia and Fournier 2009; Haga and Sakai 2012) and embryonic patterning (Friml et al. 2003). Alterations in auxin transport are associated with morphological differences both within (Bennett et al. 2006) and between (Barkoulas et al. 2008) species, and auxin has also been shown to underpin morphological convergence in leaf and rooting (Pires et al. 2013) functions. Many developmental roles of auxin transport are conserved across land plants (Cooke et al. 2002; Sanders and Langdale 2013), and disruption of transport can disrupt the development of major organ systems (Galweiler et al. 1998). These data suggest a strong potential contribution of auxin transport to morphological change in plant evolution.

The major developmentally relevant auxin transport streams occur via cell-to-cell transport. IAA is a weak acid and at the low pH of the extracellular matrix (pH 5.5), a significant fraction is protonated and can hence move passively into cells. Once inside a cell (pH 7), auxin is almost entirely deprotonated and unable to exit across the plasma membrane passively (Zazímalová et al. 2010). These considerations underlie the chemiosmotic hypothesis, which proposed polar auxin transport through tissues arises by the action of efflux proteins with polar localizations, which generate directional auxin flux (Rubery and Sheldrake 1974; Raven 1975). In line with this prediction, PIN-FORMED1 (PIN1) was identified as a basally localized auxin efflux carrier in the *Arabidopsis thaliana* shoot (Galweiler et al. 1998; Okada et al. 1991). PIN proteins have subsequently received attention due to their highly specific cellular localizations, dynamic behavior, and the striking developmental phenotypes caused by disrupting their function.

Structurally, PIN proteins appear to be secondary transporters that use the plasma membrane electrochemical gradient to drive their activity (Zazímalová et al. 2010). Previous analyses have identified a tripartite domain structure. Predicted transmembrane domains at the N- and C-termini probably form an auxin-translocation pore (Galweiler et al. 1998; Paponov et al. 2005; Krecek et al. 2009), and a central hydrophilic intracellular loop region of variable length influences protein localization patterns and activity (Dhonukshe et al. 2010; Huang et al. 2010). In *Arabidopsis*, five well-characterized proteins (PIN1-PIN4, PIN7) with long loops are plasma-membrane localized and co-ordinate many developmental processes (Benjamins and Scheres 2008). Three PINs (PIN5, PIN6, and PIN8) have shorter loops (Paponov et al. 2005; Krecek et al. 2009) and endoplasmic reticulum (ER) localization, and have been proposed to function in auxin homeostasis within cells rather than transport between cells (Mravec et al. 2009; Dal Bosco et al. 2012; Ding et al. 2012; Sawchuk et al. 2013). It has previously been suggested that this short, ER-localized type may represent the ancestral form of PIN protein (Mravec et al. 2009; Viaene et al. 2013).

To better understand the evolution of the PIN family and identify potential associations between PIN function and morphological evolution in plants, we have undertaken a phylogenetic analysis of land plant PIN proteins in conjunction with an in-depth structural analysis. We have identified 473 PIN family members, sampling to an unprecedented level within the ferns, gymnosperms, and angiosperms. Our phylogeny shows that vascular plant PINs diversified by deep duplications, but have further diversified in independent lineage-specific radiations. Our structural analysis shows that most PIN proteins have a conserved, modular loop domain, a shared “canonical” structure that dates back to the last common ancestor of all land plants. However, we show that noncanonical PINs with divergent structures have arisen from canonical precursors multiple times in the angiosperms, whereas other vascular plant groups only have canonical PINs. Our results overturn previous models of PIN protein evolution and have important ramifications for understanding structure–function relationships of PIN proteins and morphological innovation in plant evolution.

## Results

### Multiple Analyses Converge on a Similar Phylogenetic Topology

**Fig. 1.**
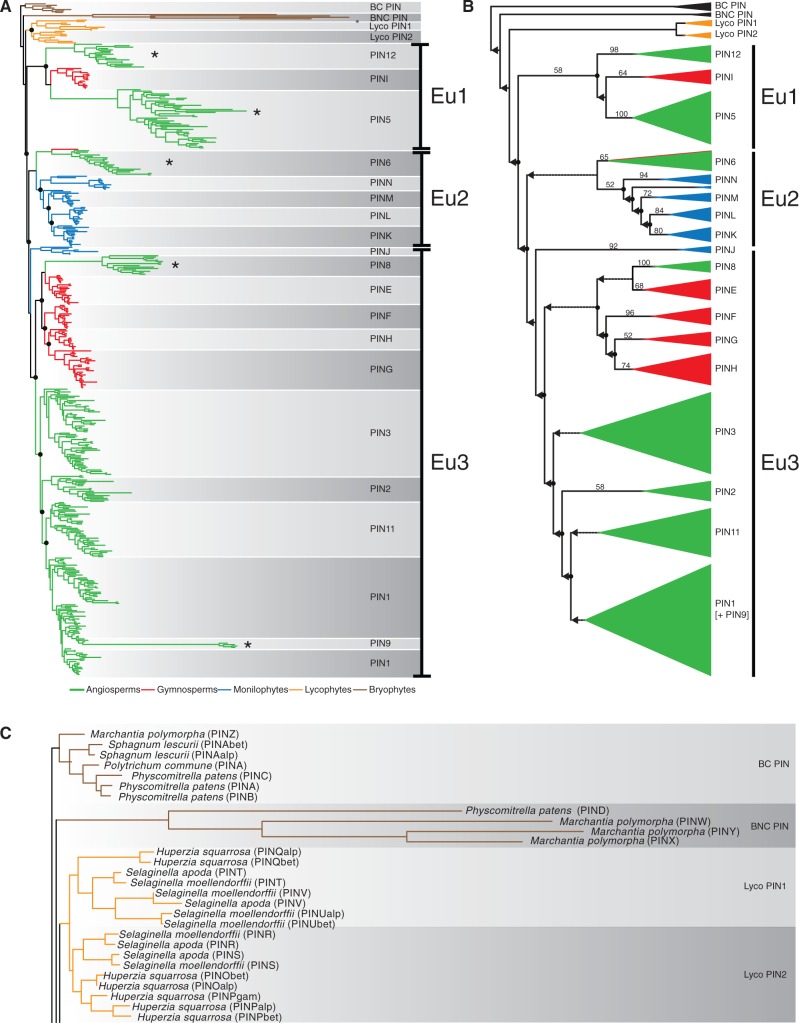
Nucleotide-level phylogenetic analysis of the PIN protein family. (*A*) Phylogram derived from ML analysis implemented in GARLI using the “total evidence” data set of 473 sequences comprising 1,809 informative characters. The tree depicts major relationships between land plant PINs and shows monophyly of vascular plant PINs. A duplication occurred within the lycophyte, and two deep euphyllophyte duplications gave rise to Eu1, Eu2, and Eu3 (gene duplications are denoted with filled black circles). Color coding: Green, angiosperms; red, gymnosperms; blue, ferns; orange, lycophytes; brown, “bryophytes.” Noncanonical PIN lineages are marked with an asterisk. BC PIN, bryophyte canonical PINs; BNC PIN, bryophyte noncanonical PINs. (*B*) Cladogram derived from ML analysis implemented in GARLI using “total evidence” data set. The tree depicts major clades of PINs across the major vascular plant lineages. Color coding: Green, angiosperms; red, gymnosperms; blue, ferns; orange, lycophytes; black, “bryophytes.” Numbers associated with internal branches denote ML bootstrap support. Branches that collapse in bootstrap analyses (<50% support) are indicated with a dotted line and leftwards-facing arrow. (*C*) Magnified view of bryophyte and lycophyte PIN clades from (*A*) showing protein types further referred to in the text.

To determine the relationships between land plant PIN proteins and evaluate the pattern of protein evolution, an alignment comprising 473 sequences from 109 species (table 1) was analyzed using “fast ML” inference at both nucleotide and amino acid levels (implemented in GARLI 2.0 and RAxML), and at the codon level (data not shown). Analyses were repeated at the nucleotide level using Bayesian approaches (implemented in MrBayes). All analyses converged on a common tree topology, revealing that the PIN phylogeny is complex with many independent gene duplication events, losses, and radiations in each major plant lineage (fig. 1 and supplementary fig. S1, Supplementary Material online). We identified at least 21 different PIN protein subclades, 15 of which are described here for the first time, and these have been named respecting existing nomenclature where possible (fig. 1).

**Table 1.** Sampling in PIN Protein Phylogenies.

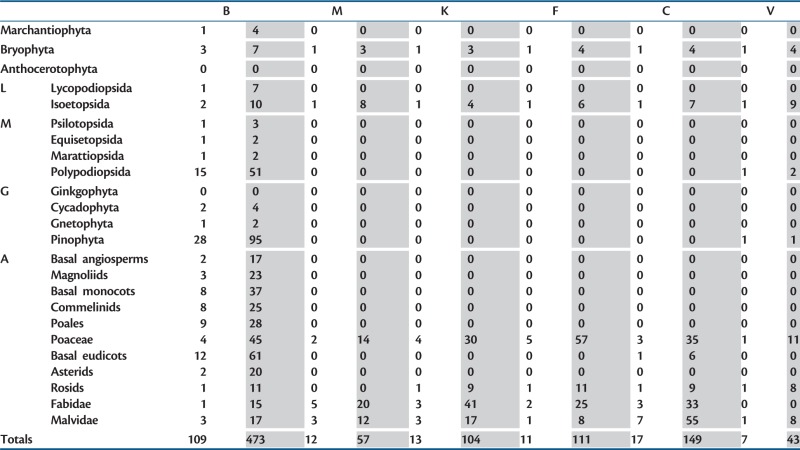

Note.—Table showing PIN gene/protein sampling rates across the plant kingdom in six studies; this study (column “B”), Mravec et al. (2009) (M), Krecek et al. (2009) (K), Forestan et al. (2012) (F), Carraro et al. (2012) (C), and Viaene et al. (2013) (V). The primary taxonomic divisions are shown at the left; lycophytes (L), monilophytes (M), gymnosperms (G), and angiosperms (A) are further broken down into major subgroups. For each study, the number of species (unshaded) and the number of sequences (shaded) obtained from each taxon are shown. Numbers for the Poaceae are shown separately from other Poales, which are in turn shown separately from other commelinids. Numbers for the Fabidae (eurosids I) and Malvidae (eurosids II) are shown separately from other rosids.

### A Bryophyte Clade Was Selected as an Outgroup

Previous reports have identified PIN proteins in charophyte sister lineages to the land plants but not in more distantly related chlorophyte algae, suggesting that PIN proteins arose in the streptophyte lineage giving rise to the land plants (Krecek et al. 2009; De Smet et al. 2011; Viaene et al. 2013). We therefore explored the possibility of using algal PIN sequences as outgroup taxa to root a land plant PIN ingroup. However, we found high divergence between algal and land plant PIN sequences, and in our analyses algal sequences were unstably placed in positions that make no sense in the context of known organismal phylogeny. We inferred that this instability arose due to long-branch artifacts and therefore restricted our sampling to the land plant PIN family. Within the bryophytes, two clades of proteins were resolved, each containing liverwort and moss sequences. As bryophytes form a basal grade in contemporary plant phylogenies (Qiu et al. 2006), we selected one of these clades (the “BC” clade; fig. 1*A* and *C*), which had a similar rate of molecular evolution to most other sequences, as an outgroup. We noted that using the alternative bryophyte PIN clade (“BNC” clade; fig. 1*A*) that has much longer branch lengths as an outgroup had no appreciable effect on relationships within a vascular plant PIN ingroup.

### Vascular Plant PINs Originated by Several Deep Duplications

**Fig. 2.**
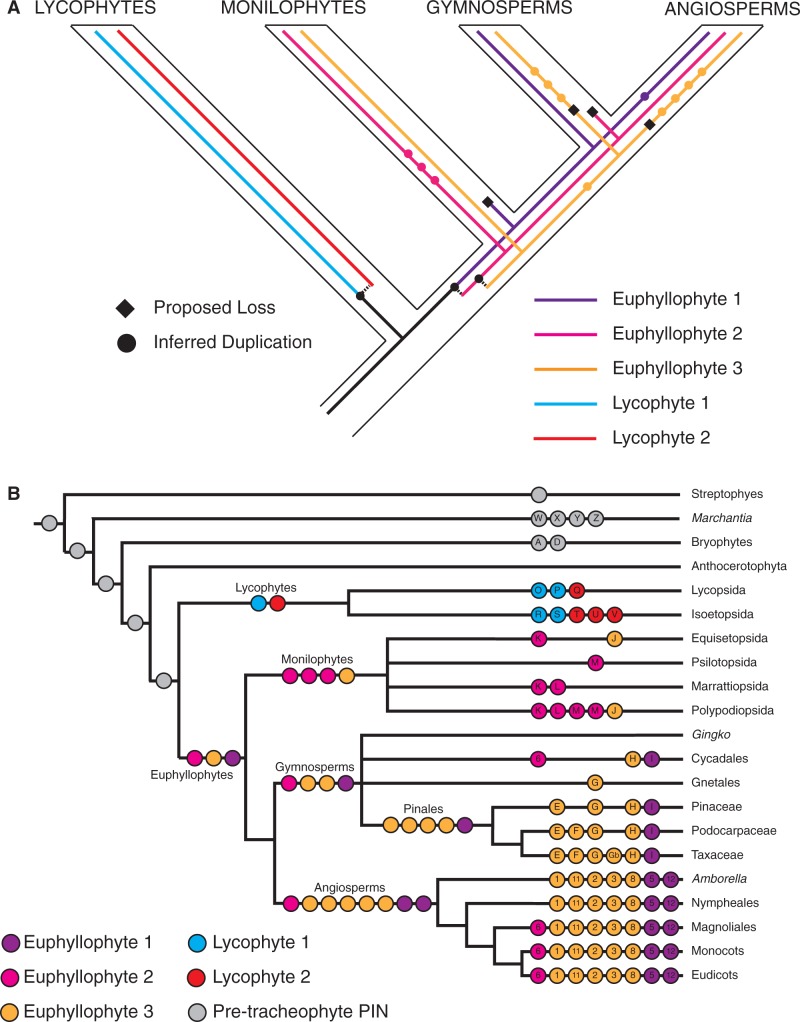
Duplication events in the PIN protein family inferred from nucleotide-level analyses. (*A*) Schematic depicting the inferred history of gene duplication and loss within the vascular plants. Inferred duplications are marked with filled circles and proposed losses are indicated with black diamonds. Paralogous lineages generated by duplication events are only shown for the three deeper-level duplications, whereas shallower duplications are marked by a filled circle. (*B*) Schematic depicting the complement of PIN proteins in major land groups. A lettered/numbered circle represents each type of PIN identified by phylogeny, and shading indicates affinities (Eu1, Eu2, and Eu3 PINs are indicated in purple, pink, and yellow, respectively, and LycoPIN1 and 2 are colored blue and red). Pretracheophyte PIN are colored in gray. Circles without symbols on internal branches represent the minimum inferred PIN protein complement in the last common ancestor of each major land plant group.

A first notable result of our analyses is that vascular plant PINs form a monophyletic group (fig. 1*A*). Within the vascular plant PIN clade, lycophyte PIN proteins form a monophyletic group with two sister lineages (“Lyco1” and “Lyco2”) that are each represented in the two main divisions of extant lycophytes (Lycopsida and Isoetopsida; figs. 1*C* and 2*B*). Euphyllophyte PINs also form a monophyletic group that is split into three main further lineages (Eu1, Eu2, and Eu3; figs. 1 and 2). These results support the hypothesis that the last common ancestor of extant vascular plants had a single PIN protein from which subsequent radiations occurred independently in lycophytes and euphyllophytes. Our results suggest that a single deep duplication occurred within the lycophytes to generate the Lyco1 and Lyco2 lineages (fig. 2*B*) and two deep duplications occurred within the euphyllophytes to give rise to the Eu1, Eu2, and Eu3 lineages. Thus, although the lycophytes had an ancestral complement of two PIN proteins, there was an ancestral complement of three for euphyllophytes (fig. 2*B*).

### Independent Radiations Occurred within Each Euphyllophyte Lineage

**Fig. 3.**
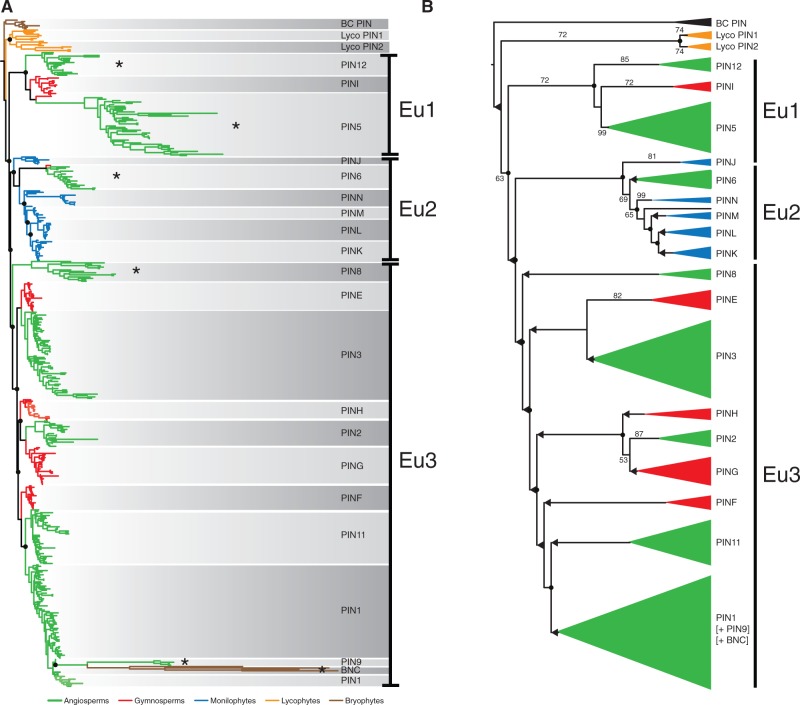
Protein-level phylogenetic analysis. (*A*) Phylogram derived from ML analysis implemented in RAxML using the “total evidence” data set of 473 sequences comprising 1,809 informative characters. The tree depicts the same major relationships as depicted in nucleotide analyses; however, the relationships among clades in the Eu3 lineages differ with four duplications inferred prior to the origin of the seed plants. Gene duplications are denoted with filled black circles. Color coding: Green, angiosperms; red, gymnosperms; blue, ferns; orange, lycophytes; brown, “bryophytes.” Noncanonical PIN lineages are marked with an asterisk. BC PIN, bryophyte canonical PINs; BNC PIN, bryophyte noncanonical PIN. (*B*) Cladogram derived from ML analysis implemented in RAxML using “total evidence” data set. Tree depicts major clades of PIN genes across the major vascular plant lineages. Color coding: Green, angiosperms; red, gymnosperms; blue, ferns; orange, lycophytes; black, “bryophytes.” Numbers associated with internal branches denote ML bootstrap support. Branches that collapse in bootstrap analyses (<50% support) are indicated with a dotted line and leftwards-facing arrow. Gene duplications are denoted with filled black circles.

The euphyllophyte lineages identified above showed major asymmetries in their subsequent evolution (fig. 2). For instance, the Eu1 lineage contains three subgroups (PIN5 and the newly identified PIN12 clades from angiosperms, and PINI from gymnosperms) in which there are multiple representatives from angiosperms but not gymnosperms. Similarly, Eu2 contains five clades, four of which are closely related proteins from monilophytes (PINK, PINL, PINM, and PINN). Monilophyte Eu2 sequences jointly form a sister group to a clade containing the PIN6 subgroup from angiosperms and a single cycad protein, the only gymnosperm representative in the Eu2 clade (fig. 1). Eu3 is the largest clade and contains 11 subgroups; one from monilophytes (PINJ), four from gymnosperms (PINE, PINF, PING, and PINH), and six from angiosperms (PIN1, PIN2, PIN3, PIN8, and the newly identified PIN9 and PIN11 clades) (O’Connor et al. 2014). These patterns suggest multiple rounds of lineage-specific duplication within Eu1, Eu2, and Eu3 (fig. 2). A significant number of losses is also required to account for current PIN complements (fig. 2*A*). In Eu1, the earliest diverging PIN lineage, we detected no monilophyte sequences. Given that Eu2 and Eu3 both contain monilophyte sequences, we infer a monilophyte loss of Eu1. Similarly, the absence of Eu2 representatives in conifers suggests an early loss of Eu2 homologs in this group of gymnosperms (fig. 2*B*). The only major incongruence between our nucleotide and protein analyses relates to the evolutionary history of Eu3. Although Eu3 incorporates the same angiosperm and gymnosperm subgroups in both analyses, the nucleotide analyses suggest gymnosperm- and angiosperm-specific radiations (fig. 1), whereas the protein analyses suggest four prespermatophyte duplication events in which the majority of the angiosperm lineages have orthologous gymnosperm clades (fig. 3). We were unable to resolve this incongruence using further analyses within Eu3 with additional characters and different coding strategies; in contrast to Eu1 and Eu2 subsets which exhibit strong support values when analyzed individually (supplementary fig. S1, Supplementary Material online), the Eu3 lineage had sporadic and low support values for key nodes. Our analyses revealed that the PIN protein complement of each major vascular plant group has a largely group-specific evolutionary history of duplication and loss.

### Robustness Analyses Support the Broad Phylogenetic Topology

**Fig. 4.**
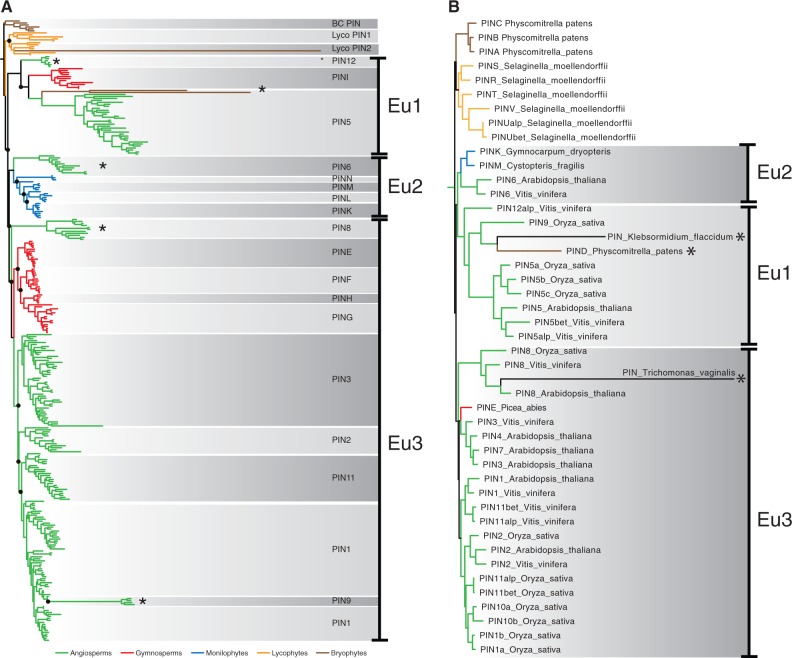
Analyses of subsets of the data to assess robustness of the total evidence data set. (*A*) Phylogram derived from ML analysis implemented in GARLI of subset of total evidence data set comprising only fragments >900 bp (∼50% of the average length of PIN genes). The topology is broadly similar the total evidence data set. A duplication gives lycophyte PINs, and two deep euphyllophyte duplications gave rise to Eu1, Eu2, and Eu3 (gene duplications are denoted with filled black circles). PINJ is missing because all fragments are <900 bp. The noncanonical long-branched bryophyte PIN clade collapses. Color coding: Green, angiosperms; red, gymnosperms; blue, ferns; orange, lycophytes; brown, “bryophytes.” Noncanonical PIN lineages are marked with an asterisk. BC PIN, bryophyte canonical PINs. (*B*) Phylogram derived from ML analysis implemented in GARLI of total evidence data set to match sampling in Viaene et al. (2013), revealing a topology that is congruent with that revealed by the total evidence data set. Note the anomalous position of algal sequences embedded in the land plants, in association with other long-branched land plant PINs in the case of *Klebsormidium*.

**Fig. 5.**
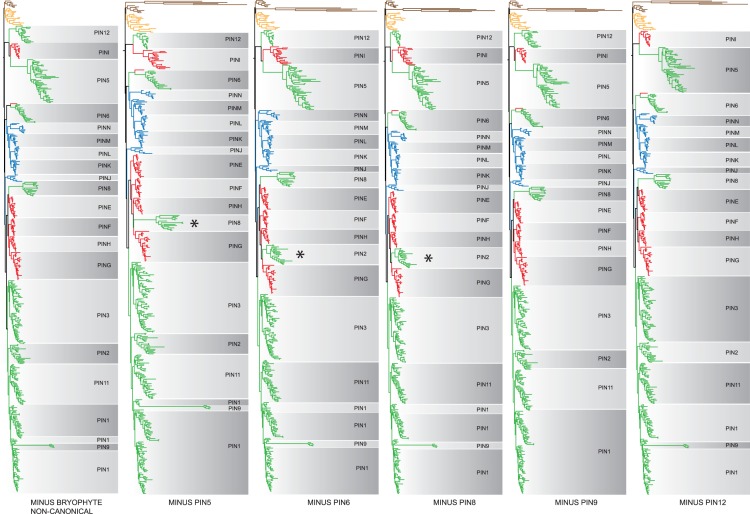
Tree topology is not an artifact of molecular rate heterogeneity. Topologies derived from multiple subsets in which selected long-branch clades have been successively removed to demonstrate that broader topological patterns are not the result of molecular rate heterogeneity. Removed clades include: NBC bryophyte lineages, PIN5, PIN6, PIN8, PIN9, and PIN12. Asterisks indicate clades whose position changes relative to that in the topology of total evidence data set. In summary, the broad topology of the tree is unaffected by the removal of the clades; however, some instabilities are revealed particularly with respect to the placement of the PIN2 and PIN8 clades. From left to right: removal of the long-branched bryophyte clade had no effect on the topology of the tree; removal of PIN5 marginally altered the placement of PIN8 relative to the gymnosperm clades E, F, G, and H; removal of PIN6 marginally altered the placement of PIN2 relative to the gymnosperm clades E, F, G, and H; removal of PIN8 marginally altered the placement of PIN2 relative to the gymnosperm clades E, F, G, and H; removal of PIN8 altered the placement of PIN2 to be sister to PIN11 and 1; and removal of PIN12 altered the placement of PIN2 to be sister to PIN1, 9, and 11. All other major relationships were unaltered by these perturbations.

The majority of the individual PIN protein clades identified in this study are supported by bootstrap analyses (fig. 1*B*). However, the larger-scale topology described above is poorly supported in bootstrap analyses, and in particular both the monophyly of vascular plant PIN proteins, and the division of euphyllophyte PIN proteins into three major lineages have poor or no statistical support at the nucleotide level and weak to no support in protein-level analyses (figs. 1*B* and 3). We therefore performed several further analyses to assess whether our conclusions are robust. Firstly, although there are some differences between the nucleotide and amino acid-level analyses (discussed further below), both analyses support the major topological divisions into Eu1, Eu2, and Eu3 (fig. 3). Second, the topology is not the product of including many fragmentary sequences because a reduced data set comprising only PIN proteins of at least 50% length (900 bp) yields an essentially identical topology (fig. 4*A*). Third, the topology is not an artifact of rapidly evolving long-branched clades of PIN genes (e.g., PIN 5, 6, 8, 9, 12, and noncanonical bryophyte lineages), because selective pruning of these long-branched clades does not alter the overall topology of the tree (fig. 5). Fourth, the topology is not a consequence of the choice of data set, since pruning our data set to match previously published data sets yielded topologies that were congruent with our findings (fig. 4*B*). Finally, when analyzed in isolation, Eu1, Eu2, and Eu3 each form strongly supported clades, and Eu1 and Eu2 have very strong support for internal relationships (supplementary fig. S1, Supplementary Material online).

### Several PIN Clades Are on Long Branches

**Fig. 6.**
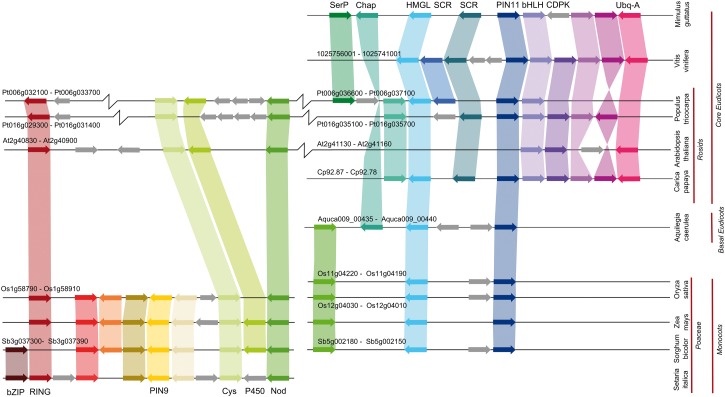
PIN9 synteny analysis. A syntenic region containing PIN9 can clearly be defined in grass species (Poaceae) (bottom left). The same syntenic region can be found in *Arabidopsis* and *Populus* (top left), but this region contains neither PIN6 or PIN12, nor indeed any PIN protein. However, in *Populus*, this syntenic region is closely linked to the location of PIN11 (top right). To test the possibility that PIN9 might have arisen from a PIN11 gene after local genomic rearrangement, we examined synteny between the PIN11 region from *Populus* and grass genomes. PIN11 syntenic regions exist in grasses and contain obvious PIN11 homologs, and are on separate chromosomes to PIN9 genes (bottom right); the fusion of these two syntenic regions (pseudo-PIN9 and PIN11) seems to be a rosid-specific event. There is no synteny between PIN9 or any other type of PIN protein. Black lines represent chromosomes, axis break symbols indicate the omission of some data for reasons of space. Where coherent genome numbering systems exist, the range of genes involved in the syntenic regions is shown. Species and phylogenetic groupings are indicated at the right-hand side. Nonsyntenic genes are indicated in gray, syntenic gene groups are each indicated in a separate color. Where gene products are known they are labeled: Cys, cystatin; Nod, nodulin; SerP, serine protease; Chap, chaperonin; HMGL, hydroxymethylglutaryl-CoA lyase; SCR, short-chain reductase; CDPK, calcium-dependent protein kinase; and Ubq-A, ubiquitin-A. Genes and distances are not shown to scale.

Our analyses revealed considerable molecular rate heterogeneity across the phylogeny, and several clades occur on long branches including the angiosperm clades PIN5, PIN6, PIN8, PIN12, *Physcomitrella patens* PIND, and *Marchantia polymorpha* PINW, PINX, and PINY. Intriguingly, our phylogeny suggests that the long-branched PIN9 clade (Wang et al. 2009) found in the grass family (Poaceae) arose from within the PIN1 clade. As the five Poaceae genomes analyzed in this study lack PIN6 and PIN12 homologs, we used synteny analysis to examine the possibility that PIN9 represents a highly divergent version of one of these genes. Our results showed that PIN9 is not syntenic with any other PIN group (fig. 6). We sampled extensively in other Poales and sister groups (Zingiberales and Areales), but did not identify any further PIN9-like sequences, suggesting that this innovation is specific to the Poaceae. Since the central loop of these proteins was largely excluded from our analysis, the higher rates of molecular evolution in these long-branched PINs are likely to reflect alterations in transmembrane domain structure.

## Canonical PIN Proteins Are Defined by a Conserved Modular Loop Structure

**Fig. 7.**
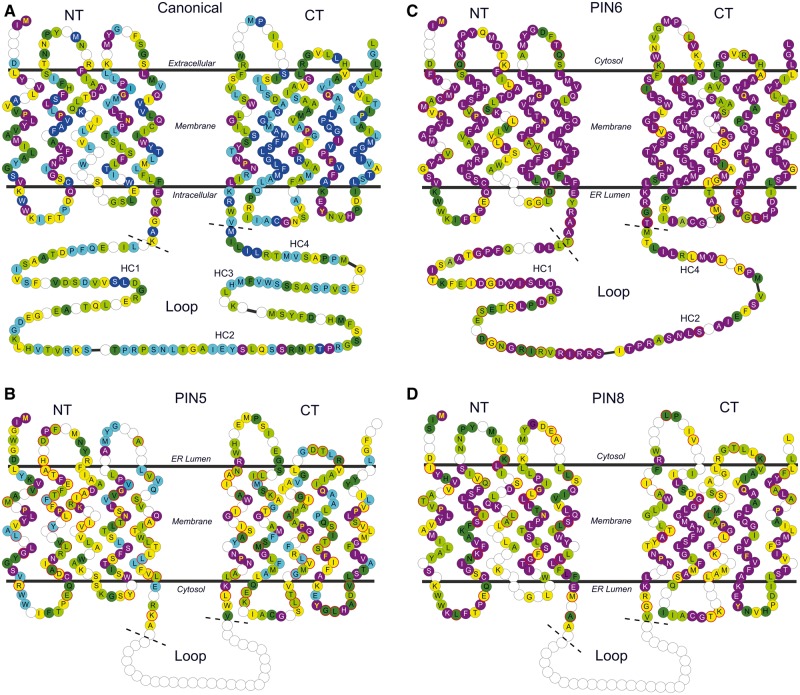
Analysis of PIN protein structure and conservation showing diagrammatic representations of topology and amino acid conservation in PIN proteins. The number and positioning of these amino acids (represented by circles) relative to both each other and the membrane are derived from our structural analysis, and represent modal predictions. The letters in the circles represent the amino acid consensus found at each position in the relevant group of PIN proteins; where no letter is present, there is no clear consensus. The color of the circle indicates the degree of amino acid identity at each position: purple, 100%; dark blue, >99%; light blue, >95%; dark green, >90%; light green, >70%; and yellow, >50%. Yellow text indicates residues that are 100% conserved in all PIN proteins. (*A*) Consensus structure of canonical PIN proteins. The four highly conserved regions of the loop found in all canonical PIN proteins are shown, the rest of the loop is omitted for clarity (black lines). (*B–D*) Consensus structures of PIN5, PIN6, and PIN8 proteins, respectively. Red circle borders indicate where a consensus amino acid in this group differs from the canonical consensus.

**Fig. 8.**
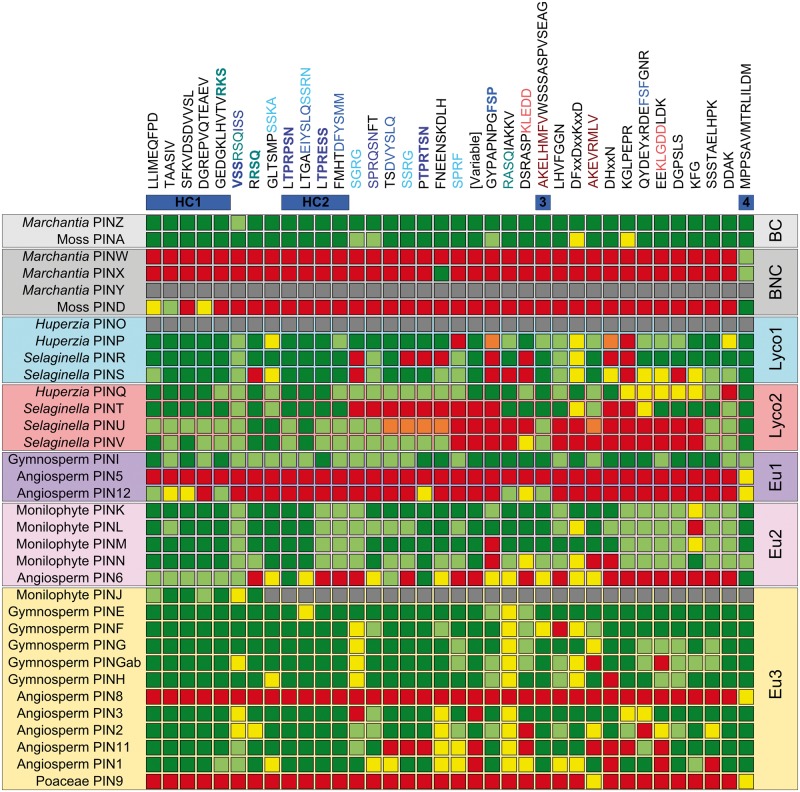
Modular loop structure in PIN proteins. Conserved motifs are shown at the top of each column in the order in which they appear within the PIN protein loop. The motifs or group of motifs that constitute the highly conserved canonical regions HC1–HC4 are shown as blue bars beneath the motifs. Motifs or submotifs known to be phosphorylated are shown in bold text. Each different type of phosphorylated motif is shown in shades of blue, along with other possible phosphorylation motifs of the same type. Other repeating motifs that are not likely to be phosphorylated, are shown in shades of red. At the left of the table, PIN protein types identified by our analysis are listed, and the major phylogenetic divisions of the family are shown at the right. Each row represents the loop structure of an individual PIN protein type, and colors indicate presence/absence of each motif in that type. Red, absent; yellow, partially present; green, present; light green, present but divergent; orange, no clear consensus; and gray, no data. In the case of “present but divergent” classification, a sequence occupies the same relative position as the given motif and has the same general structure, but contains divergent amino acids relative to the consensus sequence.

To better understand the relationship between protein structure and molecular evolution in different PIN clades, we undertook a detailed analysis of PIN protein structure. We used our alignment to systematically delineate the two predicted transmembrane domains and the central intracellular loop (hereafter simply called the “loop”). As noted in previous analyses (Krecek et al. 2009; Mravec et al. 2009; Viaene et al. 2013), we observed strong conservation at both the N- and C-termini of the protein, and less conservation in the loop. On this basis, we hypothesized that the N- and C-terminal transmembrane domains run from the start codon to the sequence “FLFEFRAAR” (or variant), and from the sequence “VWRKLIRN” (or variant) to the stop codon, respectively, with the loop in between (fig. 7). To understand the evolution of the loop, we reanalyzed its structure in detail. We observed that in a limited number of clades (D, W, X, 5, 8, and 9), the loop was relatively short (32–120 amino acids), and showed no obvious homology to the loop in any other clade; we also detected limited conservation within these clades. In all other clades for which we have data, the loop is longer (>150 amino acids), with extensive similarity within and between clades (fig. 8). By comparing these “long” clades, we found that the loop consists of a series of conserved motifs that are always arranged in the same order within the protein (fig. 8). These motifs are all present in PINZ from *M. polymorpha* (which has one of the longest loops in our data set), but in all other proteins at least some of the motifs are absent; the exact combination is specific to each clade (fig. 8). We identified four highly conserved motifs or groups of motifs that are found in the loop of almost all “long” PIN proteins (regions HC1–HC4; fig. 8), for each of which we identified a consensus sequence (fig. 7*A*). Within the conserved motifs, we identified repeated elements, many of which are known or suspected phosphorylation sites (fig. 8; Benschop et al. 2007; Huang et al. 2010; Zhang et al. 2010; Ganguly et al. 2012). The region surrounding HC2 is particularly repetitive and in many PIN proteins consists of a group of three consecutive motifs that are repeated four times (fig. 8).

Since we found that the majority of PIN proteins share this common modular loop, and in particular the regions HC1–HC4, we propose this as a canonical PIN protein structure. Hereafter, we define individual PIN proteins as canonical if they match the consensus sequence across all of HC1–HC4 with at least 50% identity or 70% similarity. By extension, we define a clade of PIN proteins as canonical if 90% of its members have canonical structure (or a clear majority in clades with fewer than ten members). This definition includes all clades except D, J, O, W, Y, X, 5, 6, 8, 9, and 12 (fig. 8). We do not have full loop sequences for PINJ, but the partial sequences covering HC1 match it with 67% identity, suggesting this is a canonical clade. In the case of PIN6 and PIN12, the loops still have homology to the canonical structure, but lack most conserved motifs, including at least one of HC1–HC4 (figs. 7*B* and 8). The clades D, W, X, 5, 8, and 9 are completely noncanonical, and we have no data for the loop of PINO or PINY (fig. 8). The terms “long” and “short” have previously been used to describe the loops of different PIN groups (e.g., Krecek et al. 2009; Viaene et al. 2013), but these terms are imprecise, and do not describe the actual structure of the loop. We suggest that in future these terms be replaced with “canonical” and “noncanonical” (and related variants), which more accurately describes structural features of the proteins. Although all canonical PIN proteins in our data set have long loops, and truly noncanonical PIN proteins have shorter loops, our definition does not preclude the possibility of either a short canonical PIN protein or a long noncanonical PIN protein. Indeed, we identified certain long PIN proteins in otherwise canonical clades that are not fully canonical by the definition provided here.

### Canonical PIN Transmembrane Domains Are very Highly Conserved

To further understand the structure of canonical PIN proteins, we analyzed their transmembrane domain structure. The length of the predicted N-terminal transmembrane domain is very consistent; in 209/269 sequences with a complete N-terminus, there are 158 amino acids (positions N1–N158; fig. 7*A*). There are 16 shorter sequences (with a minimum length of 155 amino acids), and 44 longer sequences, mostly from the Poaceae (30/44 cases), and most caused by insertion of up to 18 extra amino acids between N97 and N98 (39/44 cases). The predicted C-terminal transmembrane domain is even more consistent in length with 235/241 complete sequences having 154 amino acids (positions C1–C154; fig. 7*A*). We calculated the frequency of the most common amino acid at each core position in the transmembrane domains, across all canonical PIN proteins (fig. 7*A*). These data show that primary structure is very strongly conserved in canonical proteins, with 106 invariant or near-invariant (>99% amino acid identity) positions, and 87 showing over 90% identity (fig. 7*A*). Canonical PIN proteins thus have extensive structural conservation, and it would also be possible to define a canonical protein set based on transmembrane domain sequences; for this reason, we tentatively group PINO, but not PINY, as a canonical PIN protein. We next assessed transmembrane structure in a core set of 91 complete PIN protein sequences using the TMHMM algorithm (Sonnhammer et al. 1998); although it is generally agreed that all PIN proteins have ten helices (Krecek et al. 2009), published predictions are quite variable (Paponov et al. 2005; Mravec et al. 2009; Forestan et al. 2012). It was relatively rare that ten unambiguous helices were predicted (supplementary data set S2, Supplementary Material online), but posterior reanalysis of the hydrophobicity data (Sonnhammer et al. 1998) always revealed cryptic peaks corresponding to the “missing” helices, and we conclude that all PIN proteins are highly likely to have ten helices. The exact positions of the predicted helices within the transmembrane domains vary considerably, but using our large data set, we were able to make modal predictions for the position of each helix (supplementary data set S2, Supplementary Material online). The positions of these predicted helices fall within the boundaries of the transmembrane domains that we predicted on the basis of homology, and we therefore conclude that our delineation of domains in PIN proteins is accurate. Apart from helix 3, the predicted helices are the most highly conserved parts of the transmembrane domains (fig. 7*A*), which is unexpected since the exact amino acid composition of transmembrane helices is often unimportant (Sonnhammer et al. 1998); this probably relates to stabilization of the protein within the membrane, or function in the actual translocation of auxin across the membrane. It is also notable that, while less well conserved than the helices, the eight “minor” intra- and extracellular loops still contain several highly conserved residues, particularly the tyrosine (Y) at position C123, which is found in all PIN proteins (fig. 7*A*); important regulatory sequences may thus also be present in the minor loops.

### Noncanonical PINs Have Repeatedly Evolved by Sequence Divergence from Canonical Precursors

Noncanonical PINs formed long branches in our phylogeny, but our alignment did not contain loop sequences from these proteins, so the difference in rates of molecular evolution indicates major structural changes to the transmembrane domains. We tested the hypothesis that helix structure may be disrupted, but did not identify any major changes in length or helix position in noncanonical transmembrane domains (supplementary data set S2, Supplementary Material online). However, although we did not find any evidence of a shared noncanonical structure, we found that the primary structure of noncanonical PIN proteins strongly diverges from the canonical template (table 2). The lack of shared noncanonical structure is consistent with the homoplasious distribution of noncanonical PIN proteins across the phylogeny (fig. 1*A*, stars), and demonstrates that noncanonical structures evolved independently on at least seven occasions. As each noncanonical group is likely to have unique structural features, we compared each major noncanonical subgroup (PIN5, PIN6, PIN8, PIN9, and PIN12) with the canonical structure individually. PIN6 and PIN12 proteins closely match the canonical template at both those residues that are invariant and those that have >90% identity in canonical transmembrane domains (table 2 and fig. 7*B*). Together with the conservation of parts of the canonical loop (fig. 6), these data suggest that PIN6 and PIN12 might be best classified as “semicanonical.”

**Table 2.** Conservation of Canonical Transmembrane Domain Structure in Noncanonical PIN Proteins.

Conserved Residues in Canonical PINs		Degree/Number	Number of Which Residues also Highly Conserved in					
			NC	PIN5	PIN6	PIN8	PIN9	PIN12
	100% *n* = 56	38	42	55	42	45	54
	>99% *n* = 49	23	27	48	33	33	44
	>95% *n* = 58	14	21	46	20	29	40
	>90% *n* = 30	3	7	21	8	9	12

Note.—Highly conserved (>90% identity) positions in the transmembrane domain of canonical PIN proteins were divided into categories based on the level of amino acid identity (second column), and the number of positions in each category is shown. We assessed how many of the positions in each class were also highly conserved (>90% identity) in noncanonical PIN proteins as a group (NC), and in various noncanonical subgroups (listed in the second row); these data are enumerated in the matrix of the table.

PIN5, PIN8, and PIN9 also show some conservation at residues that are invariant in canonical PINs, but are highly divergent at all other positions (table 2 and fig. 7*C* and *D*). This variation could be due to relaxation of functional constraints or strong positive selection for new characters that modify protein function, and we reasoned that selection could be indicated by strong within-clade conservation. Accordingly, we calculated the percentage of amino acid identity at each transmembrane domain position within three angiosperm noncanonical clades (PIN5, PIN6, and PIN8) and compared it with the level of variation within a canonical clade (PIN1) sampled from the same species range. PIN1 has a very high level of self-identity (56% of positions are invariant and 76% are conserved to >90% identity; table 3), and although it varies from the canonical template, PIN6 has similar levels of self-identity, suggesting that divergent residues in PIN6 are still functionally important (table 3 and fig. 7*B*). Conversely, PIN5 and PIN8 have much lower levels of self-identity (table 3 and fig. 7*C* and *D*); only 16% of positions are invariant within the PIN5 clade and 15% of positions show no conservation at all. Thus, in noncanonical PIN proteins, the exact amino acid sequence seems to be less important than in canonical PIN proteins.

**Table 3.** Sequence Conservation in Noncanonical PIN Protein Subgroups.

Conservation	Number of Residues			
	PIN1	PIN5	PIN6	PIN8
100%	174	50	197	102
95–99%	53	39	0	0
90–95%	11	42	24	35
70–90%	39	62	44	57
50–70%	19	76	30	61
<50%	16	43	16	57
Sample size	76	42	18	15

Note.—The consistency of amino acid usage within four PIN subgroups was assessed, irrespective of the exact choice of amino acids. Each residue in the transmembrane domains was analyzed and categorized by the level of amino acid identity at that position; categories are indicated in the first column. The number of positions in each category is listed for each subgroup, along with the number of proteins in that subgroup (bottom row). The total number of positions assessed was 312 for each protein (N1–N158; C1–C154).

## Discussion

### A History of Duplications, Losses, and Paralogous Radiations

In this analysis, we have mined the rich transcriptomic resources principally generated by the 1KP project to uncover the evolutionary history of a fundamentally important class of developmental regulators—the PIN proteins. We reveal 15 new embryophyte clades of PIN proteins, again emphasizing the importance of broad taxonomic sampling for accurate reconstruction of evolutionary history. The patterns that emerge are complex and driven by ancient and recent duplications, high levels of molecular rate heterogeneity, asymmetric radiations, and losses in paralogous gene lineages. Aside from duplications in the bryophytes and lycophytes, two deep-level gene duplication events prior to the origin of the euphyllophytes generated three major lineages of PIN genes: Eu1, Eu2, and Eu3. Within each of these Eu PIN lineages, a highly asymmetric pattern of amplification and loss has generated PIN complements comprising distinct paralogs in different vascular plant groups. It is important to note that much of the tissue sampled by the 1KP project is leaf derived and inferred losses could therefore reflect shifts in PIN expression to nonleaf tissue. However, as currently depicted, four out of the five clades of the gymnosperm PIN protein complement are derived from the Eu3 lineage, and four out of five clades of the monilophyte PIN protein complement are derived from the Eu2 lineage. The only angiosperm representative of the Eu2 lineage, PIN6, is predominantly ER-localized with angiosperm-specific functions in leaf vascular development, nectary formation, and stamen development (Bender et al. 2013; Sawchuk et al. 2013). However, most monilophyte PINs are most closely related to PIN6 and consequently the cellular localization and comparative function of the many fern Eu2 PIN paralogs is intriguing. More generally, as these novel PIN clades are functionally characterized, it will be interesting to explore the degree to which PIN function is constrained by the unique evolutionary histories of these ancient paralogous lineages.

### Reconstructing the Evolutionary History of PINs Is Challenging

Analysis of such a large and diverse data set is challenging given asymmetric radiations, considerable molecular rate heterogeneity, and substantial amounts of missing data through a largely expressed sequence tag (EST)-based sampling method. Despite poor statistical support at deep nodes, we have rigorously tested the topology with numerous analyses (figs. 3–5), and are confident that the main findings of this study will hold for the following reasons. Firstly, within individual PIN clades, the order of branching closely matches known organismal phylogenies suggesting that the data are accurately recovering phylogenetic relationships. Secondly, the backbone arrangement and phylogenetic order of Eu1, Eu2, and Eu3 lineages are retrieved in analyses at both the nucleotide and protein levels (figs. 1 and 3). Thirdly, analyzing Eu1, Eu2, and Eu3 as separate data sets reveals strong support for relationships, at least within Eu1 and Eu2 (supplementary fig. S1, Supplementary Material online). Fourthly, the overall topology is not perturbed by significant changes in the data set, such as the removal of partial sequences (fig. 4*A*) or pruning of long-branched clades (fig. 5). Finally, as discussed below, our analysis is similar in key details to most previously phylogenies, and we recover the same essential relationships when we apply our methodology to similar taxon-poor data sets (fig. 4*B*) We conclude that despite low statistical support, the backbone topology is remarkably robust to perturbation by various forms of subsampling.

### Our Phylogeny Is Congruent with Previous Phylogenies

Previously published PIN family phylogenies have all used similar sampling strategies, primarily comprising sequences from angiosperm species (clustered into the grasses and rosids) and sequences from *Ph. patens* and *Selaginella moellendorffii* as sole or main representatives of nonangiosperm diversity (table 1) (Krecek et al. 2009; Mravec et al. 2009; Carraro et al. 2012; Forestan et al. 2012; Viaene et al. 2013). These analyses were mainly protein-based reconstructions, using neighbor-joining, Bayesian or ML approaches, and giving rise to two basic topologies. The first topology (A) has canonical *Physcomitrella* and *Selaginella* PINs arranged as two basal clades, with canonical angiosperm PINs forming a clade that is either sister to a noncanonical clade or emerges from a noncanonical grade between lycophyte and angiosperm canonical PINs (Krecek et al. 2009; Mravec et al. 2009; Carraro et al. 2012). The second topology (B) has all canonical PIN proteins plus PIN6 forming a single clade, with noncanonical PIN proteins arranged as a basal grade (Forestan et al. 2012; Viaene et al. 2013). Although topology A reflects organismal phylogeny and suggests lineage-specific PIN diversification, topology B is essentially a direct reflection of similarities in protein structure. Our phylogeny supports topology A and is closely congruent in broad topology to the phylogeny of Carraro et al. (2012) who also used ML analyses based on DNA sequences. Where our analysis markedly differs from previous analyses is that it introduces many previously undetected clades from gymnosperms and monilophytes, and thereby provides context for the evolution of noncanonical PIN proteins from angiosperms. These effects are primarily a result of massively increased sampling outside the angiosperms (table 1), and are not due to the particular methodology used in this study. Indeed, many of the medium-level phylogenetic relationships we observed are also supported in previous analyses. For instance, the placement of the noncanonical PIN8 and PIN9 clades with the large angiosperm canonical PIN clade is supported by the analysis of Carraro et al. (2012), whereas the affinity of PIN6 with PINM/PINL from monilophytes was suggested by Viaene et al. (2013). Our division of euphyllophyte PINs into three large lineages is a novel result and it should be noted that if the gymnosperm and monilophyte sequences are removed, the topology of our phylogeny would collapse into a grade of PIN5, PIN6, and PIN8, as observed in previous analyses. Our results show the advantages dense and evenly dispersed taxonomic sampling.

### Relationships within the Eu3 Lineage Are Problematic

Despite these advantages, relationships within the Eu3 lineage remain problematic. Although all analyses agree that Eu3 includes gymnosperm clades PINE, F, G, and H and angiosperm clades PIN1, 2, 3, 9, and 11 (figs. 1 and 3), the protein analyses erroneously place the bryophyte noncanonical PIN genes with the monocot-specific PIN9 clade (a clear case of long branch attraction) and also place the monilophyte clade PINJ at the base of Eu2 (without bootstrap support) (fig. 3). Although the nucleotide analyses suggest gymnosperm and angiosperm-specific radiations (fig. 1), the protein analyses suggest four prespermatophyte duplication events in which the majority of the angiosperm lineages have orthologous gymnosperm clades (fig. 3). These alternative protein and nucleotide-based scenarios clearly have different implications for the evolution of PIN structures and functions in Eu3, and structural features of the loop in different Eu3 PIN clades did not allow us to discriminate between these scenarios. There are no obvious shared-derived loop structures between the gymnosperm and angiosperm pairs that are implied in the protein-level analysis (e.g., PIN3 and PINE; fig. 3), so even if they shared a recent common ancestor, these pairs have subsequently followed different evolutionary trajectories (fig. 8). Equally, there are no clear shared-derived structural similarities in the loop that support the large gymnosperm and angiosperm groupings proposed by the nucleotide-level analysis (figs 1 and 8). In general, angiosperm PINs have shorter loops than the gymnosperm PINs, and the protein-level tree would require multiple convergent losses of the same motifs in the angiosperm Eu3 lineages (fig. 8). This is not an implausible scenario; for instance, perhaps a regulatory protein that interacts with those motifs was lost early in angiosperm evolution. The topological uncertainty surrounding the Eu3 clade will probably not be resolved without additional sampling, particularly in early diverging gymnosperm lineages.

### Land Plant PIN Proteins Probably Evolved from a Single Canonical Ancestor

Our analysis clearly demonstrates that there is a highly conserved canonical PIN structure represented in all plant groups, and we therefore infer that the last common ancestor of land plants had at least one canonical PIN protein. On a strict reading of our phylogeny, the grouping of PIND from *Ph. patens* with PINW, PINX, and PINY from *Marchantia* (fig. 1, “BNC”) would seem to imply a second, noncanonical PIN lineage in basal land plants. However, there is no shared-derived structural similarity between any of these highly divergent proteins. Taken together, the canonical nature of all PIN clades in gymnosperms and monilophytes (including those which are sister the noncanonical PIN5, PIN8, PIN6, and PIN12 clades from angiosperms), the lack of structural similarity between noncanonical protein types, and the inferred monophyly of vascular plant PINs all strongly suggest that there is not a unified noncanonical lineage arising from the last common ancestor of land plants. We therefore postulate that PIND, PINX, PINW, and PINY group together because of long-branch attraction, that these proteins evolved independently from canonical ancestors, and that there was single canonical PIN protein in the last common ancestor of all land plants. As further genomic resources become available, increased sampling of bryophyte PIN proteins should help to resolve this issue.

Although it has previously been proposed that the “short” proteins with an ER-localization are the ancestral form of PIN proteins in land plants (Mravec et al. 2009; Viaene et al. 2013), our results clearly demonstrate that canonical “long” PIN proteins were one ancestral form. It has previously been suggested that the last common ancestor of land plants had one “long” and one “short” PIN protein, but that the algal ancestors of land plants had only “short” PIN proteins (Viaene et al. 2013). In our view, there are currently too few data available to make conclusions about the structure and evolution of algal PIN proteins, and those sequences that are available are neither canonical nor truly noncanonical in structure. An ancestral ER-localization was previously inferred for land plant PIN proteins based on localization of PINA from *Ph. patens* to the ER in tobacco BY-2 cells (Mravec et al. 2009), but there are no functional data to support this localization. Our data do not directly address the issue of cellular localization, but given the very high structural conservation between characterized proteins and other canonical PINs, we speculate that in general canonical PINs will be plasma-membrane localized.

### Noncanonical PIN Proteins May be Neofunctional

The high degree of structural conservation in canonical PINs suggests that strong selection maintains their function as auxin carriers. This in turn raises intriguing questions about the structural divergence seen in noncanonical PIN proteins. Although highly divergent, the noncanonical structures have been maintained over considerable evolutionary distance. For example, PIN5, PIN6, and PIN8 show little evidence of gene loss, indicating that they are not simply in various stages of pseudogenization. PIN12 is also well conserved in basal angiosperms but has been lost in many core eudicots, suggesting that its function may have become obsolete. In the semicanonical PIN6 clade (and probably in PIN12), the retention of many of the highly conserved transmembrane domain residues suggests that tertiary structure of the transmembrane pore might be very similar to canonical PIN proteins, so these proteins are probably still selective auxin carriers, albeit with divergent localizations and activities consistent with their divergent loop structure (Sawchuk et al. 2013). However, for other noncanonical PIN proteins, the much smaller set of conserved transmembrane domain residues and increased variability in other positions makes it very likely that these proteins have divergent functions from canonical PIN proteins. One intriguing possibility is that noncanonical PINs are broader spectrum carriers for auxin-like molecules, including auxin conjugates. Consistent with this hypothesis, PIN8 seems able to transport both indole butyric acid and the auxin analog 2,4-D (normally considered a poor substrate for PIN proteins; Ding et al. 2012), and given the ER-localization of PIN5 and PIN8 a function in the homeostatic partitioning of auxin metabolites is an attractive possibility.

### Canonical PIN Proteins May Have Subfunctionalized by Modifications to the Loop

Motifs in the intracellular loop in PIN proteins can mediate regulation of PIN protein activity and localization in *Arabidopsis*. For instance, the repeated motif TPRXSS/N is phosphorylated by the PINOID protein kinase family, and may contribute to the localization of PINs (Dhonukshe et al. 2010; Huang et al. 2010). One of the most striking aspects of our analysis is that the most complete loop structures are seen in PINZ from *Marchantia* and PINA from mosses, which represent the sole types of canonical PIN proteins in these lineages. In contrast, the majority of canonical PINs from vascular plants lack a subset of loop motifs; the exact combination of motifs present is specific to the protein type, but if the entire canonical PIN complement in each species is considered together, almost all motifs are represented (fig. 8). This pattern of evolution suggests that as numbers of canonical PIN proteins increased, loop motifs were partitioned into different proteins, conferring a different subset of regulatory inputs to each protein. We therefore propose that canonical PIN proteins subfunctionalized by selective loss of loop motifs. The advantages of such specialization are clear; the generalized canonical PIN proteins in basal groups can probably perform several roles depending on their cellular context, but vascular plant proteins with specialized functions can perform different roles in the same cellular context. Our hypothesis is supported by evidence from angiosperms showing that different PIN proteins in the same cell can have different localizations (Wisniewska et al. 2006). The variant expression patterns of closely related PIN proteins such as PIN3, PIN4, and PIN7 suggest that changes in expression pattern are also likely to contribute to subfunctionalization (Blilou et al. 2005).

### PIN Protein Diversification Has Implications for Studies of Morphological Evolution

Our structural analyses suggest that the role of PIN proteins as auxin transporters is conserved within the land plants. By precisely regulating the auxin distribution, PIN proteins provide spatial specificity to downstream signaling pathways, and protein structure contributes to PIN localization (Dhonukshe et al. 2010; Huang et al. 2010). These results have important implications for understanding the evolution of morphological novelty, as illustrated by a consideration of leaf evolution. As leaves evolved independently in the diploid shoot systems of angiosperms, monilophytes, lycophytes, and the haploid shoots of mosses and liverworts (Langdale and Harrison 2008), the pattern of initiation in each group is distinct. Development ranges from iterative recruitment of a pool of cells from several cell layers in angiosperms (Scarpella et al. 2010) to recruitment of one or two epidermal cells to development in lycophytes (Harrison et al. 2007), and mosses (Harrison et al. 2009). Despite such divergent initiation patterns, each of these processes is regulated by auxin transport (Reinhardt et al. 2000; Sanders and Langdale 2013), and our data indicate that structurally divergent paralogous PIN proteins could well contribute to development.

## Materials and Methods

### Bioinformatic Retrieval of PIN Genes

*PIN* cDNA sequences were identified from previously published analyses together with BLAST searches from four primary sources: Phytozome (www.phytozome.net, last accessed May 1, 2014), the Ancestral Angiosperm Genome Project (www.ancangio.uga.edu, last accessed May 1, 2014), NCBI Blast, and the 1KP project (www.onekp.com, last accessed May 1, 2014). Accession numbers are listed in supplementary data set S3, Supplementary material online. *Arabidopsis PIN1* or *PIN5* sequences were judged at the outset to represent the broad diversity of *PIN* genes and used as the search sequences employing the tBlastX option. For nonannotated sequences derived from EST data sets, translations across all six reading frames were searched for significant ORFs, and the longest open reading frame (ORF) extracted for alignment. Very short sequences (<100 amino acids) were generally discarded. Where two or more partial sequences from the same species were independently assigned to the same subgroup by initial phylogenetic analyses and exhibited significant sequence overlap, the sequences were scaffolded into a single consensus sequence to reduce the overall number of sequences in the data set. These are clearly marked in supplementary data set S3, Supplementary material online. A total of 370 unique PIN sequences from the 1KP database were deposited in GenBank (accession numbers: KJ664232–KJ664532).

### Alignment

All alignments were performed at the amino acid level. Initially, only full-length protein sequences from completed genomes were used to build a preliminary alignment. A total of 96 PIN protein sequences from the genomes of *A. thaliana*, *Populus trichocarpus*, *Vitis vinifera*, *Solanum lycopersicum*, *Oryza sativa*, *Sorghum bicolor*, *Zea mays*, *Brachypodium distachyon* (all angiosperms), *Se. moellendorffii* (lycophyte), and *Ph. patens* (moss) were identified for this purpose (supplementary data set S3, Supplementary Material online). These sequences were aligned using with MAFFT, using an E-INS-I alignment strategy (mafft.cbrc.jp/alignment/software/, last accessed May 1, 2014). The alignment was further manually refined using the software program Se-Al (tree.bio.ed.ac.uk/software/seal/, last accessed May 1, 2014). This initial alignment was subsequently expanded through the addition of partial cDNA from a variety of EST databases, in particular from the 1KP project (www.onekp.com, last accessed May 1, 2014) (supplementary data set S3, Supplementary Material online). These sequences have a variety of length and coverage, but our initial full-length alignment provided a scaffold, which allowed them to be incorporated into the alignment. We added the new sequences in by hand in order to produce an optimal alignment (supplementary data set S5*A*, Supplementary Material online).

Although alignments were previously aligned by codon, phylogenetic analyses were performed at the nucleotide level. The N- and C-termini of the proteins were well aligned but an overall lack of conservation in the center of the proteins resulted in generally poorer alignment. Regions that could not be confidently aligned at the amino acid level were excluded from the analysis. To determine the final exclusion parameters, the alignment was subject to reiterative preliminary analyses to explore the effect of including different parts. Trees derived from these preliminary analyses were examined to determine: 1) The extent to which the tree topology is robust to variable alignment; 2) the extent to which different alignments generate the same topology regardless of the tree building optimality criterion; and 3) the degree to which the topology tracks organismal angiosperm phylogeny within paralogous clades. In evaluating the performance of these exploratory analyses, we approached a robust optimized alignment that was selected for final analyses. Gaps and missing ends of partial sequences and incomplete ESTs were coded as missing data. The final alignment included 473 taxa, with 1,809 nt of which 1,690 were parsimony informative, with 45% missing data. (analyzed alignment in supplementary data set S5*B*, Supplementary Material online).

### Phylogenetic Analyses

The final alignment was analyzed using PartitionFinder (Lanfear et al. 2012) to select the best-fit partitioning schemes and to choose among models of molecular evolution at both the nucleotide and amino acid level. Explored partition schemes included the three separate codon positions, and the N-terminus, C-terminus, and intracellular loop sections. In all instances, PartitionFinder (Lanfear et al. 2012) suggested analyzing the partitions separately under a GTR + I + G model for nucleotides according to the AIC and BIC selection criteria. The protein analyses were run as a single partition under the JTT + I + G substitution model chosen according to the AIC and BIC selection criteria. All partitions schemes were then further analyzed by “fast ML” analysis to explore the effect of partitioning on tree topology. For nucleotide-level ML analyses, we employed the program GARLI (Genetic Algorithm for Rapid Likelihood Inference; version 2.0) (Zwickl 2006). Analyses were run with default options, except that the “significanttopochange” parameter was reduced to 0.01 to make searches more stringent. ML bootstrap analyses were conducted with the default parameters and 500 replicates. We performed 100 replicate GARLI analyses and selected the topology with the highest likelihood score. Similarly, codon-level analyses are performed in GARLI using an empirical + F, 6-rate model, with 12 replicate analyses and 100 bootstrap repetitions. For the protein-level analyses, we employed the program RAxML with 1,000 fast bootstrap replicates. Bayesian analyses were implemented in MrBayes 3.2.2 (Ronquist et al. 2012) with a GTR + I + G model of evolution, and 5 million generations, with two hot and two cold chains, and burn-in of 25%. Convergence was assessed at standard deviation of 0.01. Posterior probabilities were derived from a majority rule consensus over the final 1 million generations of post burn-in trees.

### Assessing the Robustness of the Phylogeny

Although the majority of clades are supported in these analyses, the backbone of the tree is prone to collapse in bootstrap analyses. We reasoned that the placement of several of the longer-branched PIN clades (PIN5, PIN6, PIN8, PIN9, and PIN12) might become unstable during bootstrap subsampling and so we performed a series of analyses on data partitions in which the longer-branched clades were selectively pruned, to explore the effect of these longer branches on overall tree topology (not shown). We then evaluated the topology and bootstrap support on the trees derived from these pruned subsets. These subsets included a data set pruned to match earlier taxon-poor analyses (Viaene et al. 2013), a subset excluding PIN5, a subset excluding PIN6, a subset excluding PIN9, and a subset excluding the noncanonical “Bryophyte” PIN genes. Finally, we also tested the effect of missing data by performing an analysis on a subset of the data set that comprised only sequences of at least 50% average PIN length, that is, over 900 bp (missing data 38.5%). Individual lineages Eu1, Eu2, and Eu3 were also further analyzed at the nucleotide level using both ML and Bayesian approaches.

### Outgroup Designation

A variety of outgroup options were explored to root the phylogenetic analyses: the PIN-like (PILS) proteins (Barbez et al. 2012), charophyte PIN proteins, and canonical “bryophyte” PIN proteins. The PIN-like proteins proved too divergent from PIN proteins to allow adequate alignment and use as outgroup sequences. Likewise, Charophyte PIN proteins were highly divergent and in the context of preliminary phylogenetic analyses were placed on exceptionally long branches and often appeared within the “ingroup,” nested within angiosperm PIN sequences in positions that were unstable and clearly erroneous. The canonical bryophyte PIN proteins were consistently placed as a monophyletic clade outside of a tracheophyte ingroup consistent with contemporary concepts of land plant phylogeny (Qiu et al. 2006). Consequently, the canonical bryophyte PINs were used as an outgroup for rooting phylogenetic relationships among the tracheophyte PIN lineages, and all subset analyses.

## Supplementary Material

Supplementary figure S1 and data sets S2, S3, and S5*A* and *B* are available at *Molecular Biology and Evolution* online (http://www.mbe.oxfordjournals.org/).

Supplementary Data
