# Supplementary material for: Paralogous Radiations of PIN Proteins with Multiple Origins of Noncanonical PIN Structure
Source: Mol Biol Evol. 2014 Apr 23;31(8):2042–60. doi: 10.1093/molbev/msu147 (PMC4104312; doi:10.1093/molbev/msu147)
Supplement: Supplementary Data [file supp_31_8_2042__index.html]

Paralagous radiations of PIN proteins with multiple origins of non-canonical PIN structure — Paralogous Radiations of PIN Proteins with Multiple Origins of Noncanonical PIN Structure — Paralogous Radiations of PIN Proteins with Multiple Origins of Noncanonical PIN Structure — Supplementary Data 

# Paralogous Radiations of PIN Proteins with Multiple Origins of Noncanonical PIN Structure

## Supplementary Data

files

**Files in this Data Supplement:**

- Supplementary Data - pdf file
- Supplementary Data - docx file
- Supplementary Data - docx file
- Supplementary Data - nex file
- Supplementary Data - nex file
