# Supplementary material for: Paralogous Radiations of PIN Proteins with Multiple Origins of Noncanonical PIN Structure
Source: Mol Biol Evol. 2014 Apr 23;31(8):2042–60. doi: 10.1093/molbev/msu147 (PMC4104312; doi:10.1093/molbev/msu147)
Supplement: Supplementary Data [file supp_msu147_Supplementary_Figure_1.pdf]

Eu3

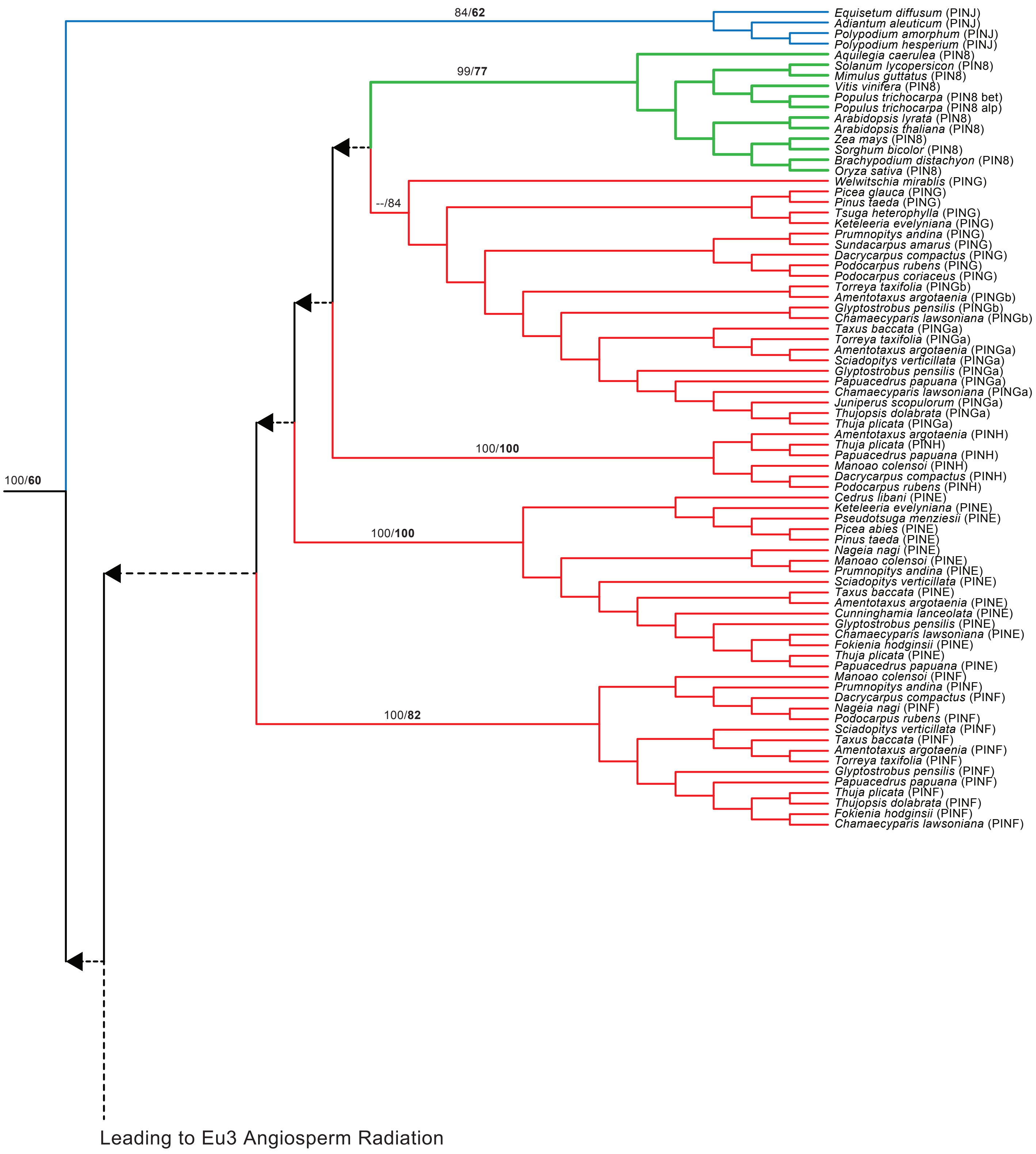

Additional Figure 1: Analyses of individual lineages Eu1, Eu2, Eu3, Cladograms depicting topologies derived from GARLI analyses (analyses conducted with bryophyte and lycophyte outgroups but outgroups not shown). Support values shown for the major nodes, absent values for major nodes indicate no support. ML bootstrap support values on the left, posterior probabilities derived from bayesian analyses on the right in bold.

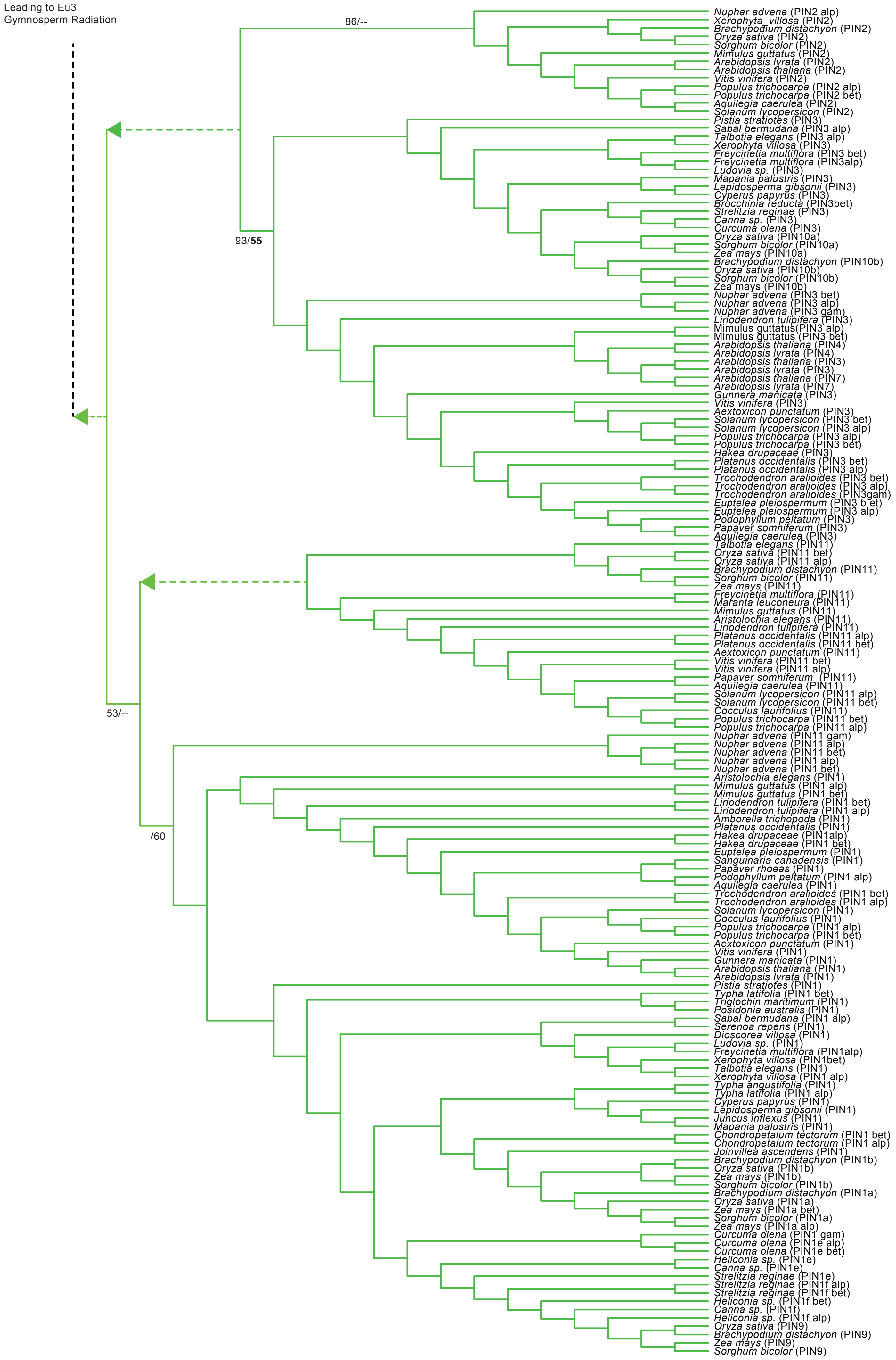

Leading to Eu3
Gymnosperm Radiation
86/--
93/55
53/--
--/60
Nuphar advena (PIN2 alp)
Xerophyta villosa (PIN2)
Brachypodium distachyon (PIN2)
Oryza sativa (PIN2)
Sorghum bicolor (PIN2)
Mimulus guttatus (PIN2)
Arabidopsis lyrata (PIN2)
Arabidopsis thaliana (PIN2)
Vitis vinifera (PIN2)
Populus trichocarpa (PIN2 alp)
Populus trichocarpa (PIN2 bet)
Aquilegia caerulea (PIN2)
Solanum lycopersicon (PIN2)
Pistia stratiotes (PIN3)
Sabal bermudana (PIN3 alp)
Talbotia elegans (PIN3 alp)
Xerophyta villosa (PIN3)
Freycinetia multiflora (PIN3 bet)
Freycinetia multiflora (PIN3alp)
Ludovia sp. (PIN3)
Mapania palustris (PIN3)
Lepidosperma gibsonii (PIN3)
Cyperus papyrus (PIN3)
Brocchinia reducta (PIN3bet)
Strelitzia reginae (PIN3)
Canna sp. (PIN3)
Curcuma olena (PIN3)
Oryza sativa (PIN10a)
Sorghum bicolor (PIN10a)
Zea mays (PIN10a)
Brachypodium distachyon (PIN10b)
Oryza sativa (PIN10b)
Sorghum bicolor (PIN10b)
Zea mays (PIN10b)
Nuphar advena (PIN3 bet)
Nuphar advena (PIN3 alp)
Nuphar advena (PIN3 gam)
Liriodendron tulipifera (PIN3)
Mimulus guttatus(PIN3 alp)
Mimulus guttatus (PIN3 bet)
Arabidopsis thaliana (PIN4)
Arabidopsis lyrata (PIN4)
Arabidopsis thaliana (PIN3)
Arabidopsis lyrata (PIN3)
Arabidopsis thaliana (PIN7)
Arabidopsis lyrata (PIN7)
Gunnera manicata (PIN3)
Vitis vinifera (PIN3)
Aextoxicon punctatum (PIN3)
Solanum lycopersicon (PIN3 bet)
Solanum lycopersicon (PIN3 alp)
Populus trichocarpa (PIN3 alp)
Populus trichocarpa (PIN3 bet)
Hakea drupaceae (PIN3)
Platanus occidentalis (PIN3 bet)
Platanus occidentalis (PIN3 alp)
Trochodendron aralioides (PIN3 bet)
Trochodendron aralioides (PIN3 alp)
Trochodendron aralioides (PIN3gam)
Euptelea pleiospermum (PIN3 b et)
Euptelea pleiospermum (PIN3 alp)
Podophyllum peltatum (PIN3)
Papaver somniferum (PIN3)
Aquilegia caerulea (PIN3)
Talbotia elegans (PIN11)
Oryza sativa (PIN11 bet)
Oryza sativa (PIN11 alp)
Brachypodium distachyon (PIN11)
Sorghum bicolor (PIN11)
Zea mays (PIN11)
Freycinetia multiflora (PIN11)
Maranta leuconeura (PIN11)
Mimulus guttatus (PIN11)
Aristolochia elegans (PIN11)
Liriodendron tulipifera (PIN11)
Platanus occidentalis (PIN11 alp)
Platanus occidentalis (PIN11 bet)
Aextoxicon punctatum (PIN11)
Vitis vinifera (PIN11 bet)
Vitis vinifera (PIN11 alp)
Papaver somniferum (PIN11)
Aquilegia caerulea (PIN11)
Solanum lycopersicon (PIN11 alp)
Solanum lycopersicon (PIN11 bet)
Cocculus laurifolius (PIN11)
Populus trichocarpa (PIN11 bet)
Populus trichocarpa (PIN11 alp)
Nuphar advena (PIN11 gam)
Nuphar advena (PIN11 alp)
Nuphar advena (PIN11 bet)
Nuphar advena (PIN1 alp)
Nuphar advena (PIN1 bet)
Aristolochia elegans (PIN1)
Mimulus guttatus (PIN1 alp)
Mimulus guttatus (PIN1 bet)
Liriodendron tulipifera (PIN1 bet)
Liriodendron tulipifera (PIN1 alp)
Amborella trichopoda (PIN1)
Platanus occidentalis (PIN1)
Hakea drupaceae (PIN1alp)
Hakea drupaceae (PIN1 bet)
Euptelea pleiospermum (PIN1)
Sanguinaria canadensis (PIN1)
Papaver rhoeas (PIN1)
Podophyllum peltatum (PIN1 alp)
Aquilegia caerulea (PIN1)
Trochodendron aralioides (PIN1 bet)
Trochodendron aralioides (PIN1 alp)
Solanum lycopersicon (PIN1)
Cocculus laurifolius (PIN1)
Populus trichocarpa (PIN1 alp)
Populus trichocarpa (PIN1 bet)
Aextoxicon punctatum (PIN1)
Vitis vinifera (PIN1)
Gunnera manicata (PIN1)
Arabidopsis thaliana (PIN1)
Arabidopsis lyrata (PIN1)
Pistia stratiotes (PIN1)
Typha latifolia (PIN1 bet)
Triglochin maritimum (PIN1)
Posidonia australis (PIN1)
Sabal bermudana (PIN1 alp)
Serenoa repens (PIN1)
Dioscorea villosa (PIN1)
Ludovia sp. (PIN1)
Freycinetia multiflora (PIN1alp)
Xerophyta villosa (PIN1bet)
Talbotia elegans (PIN1)
Xerophyta villosa (PIN1 alp)
Typha angustifolia (PIN1)
Typha latifolia (PIN1 alp)
Cyperus papyrus (PIN1)
Lepidosperma gibsonii (PIN1)
Juncus inflexus (PIN1)
Mapania palustris (PIN1)
Chondropetalum tectorum (PIN1 bet)
Chondropetalum tectorum (PIN1 alp)
Joinvillea ascendens (PIN1)
Brachypodium distachyon (PIN1b)
Oryza sativa (PIN1b)
Zea mays (PIN1b)
Sorghum bicolor (PIN1b)
Brachypodium distachyon (PIN1a)
Oryza sativa (PIN1a)
Zea mays (PIN1a bet)
Sorghum bicolor (PIN1a)
Zea mays (PIN1a alp)
Curcuma olena (PIN1 gam)
Curcuma olena (PIN1e alp)
Curcuma olena (PIN1e bet)
Heliconia sp. (PIN1e)
Canna sp. (PIN1e)
Strelitzia reginae (PIN1e)
Strelitzia reginae (PIN1f alp)
Strelitzia reginae (PIN1f bet)
Heliconia sp. (PIN1f bet)
Canna sp. (PIN1f)
Heliconia sp. (PIN1f alp)
Oryza sativa (PIN9)
Brachypodium distachyon (PIN9)
Zea mays (PIN9)
Sorghum bicolor (PIN9)

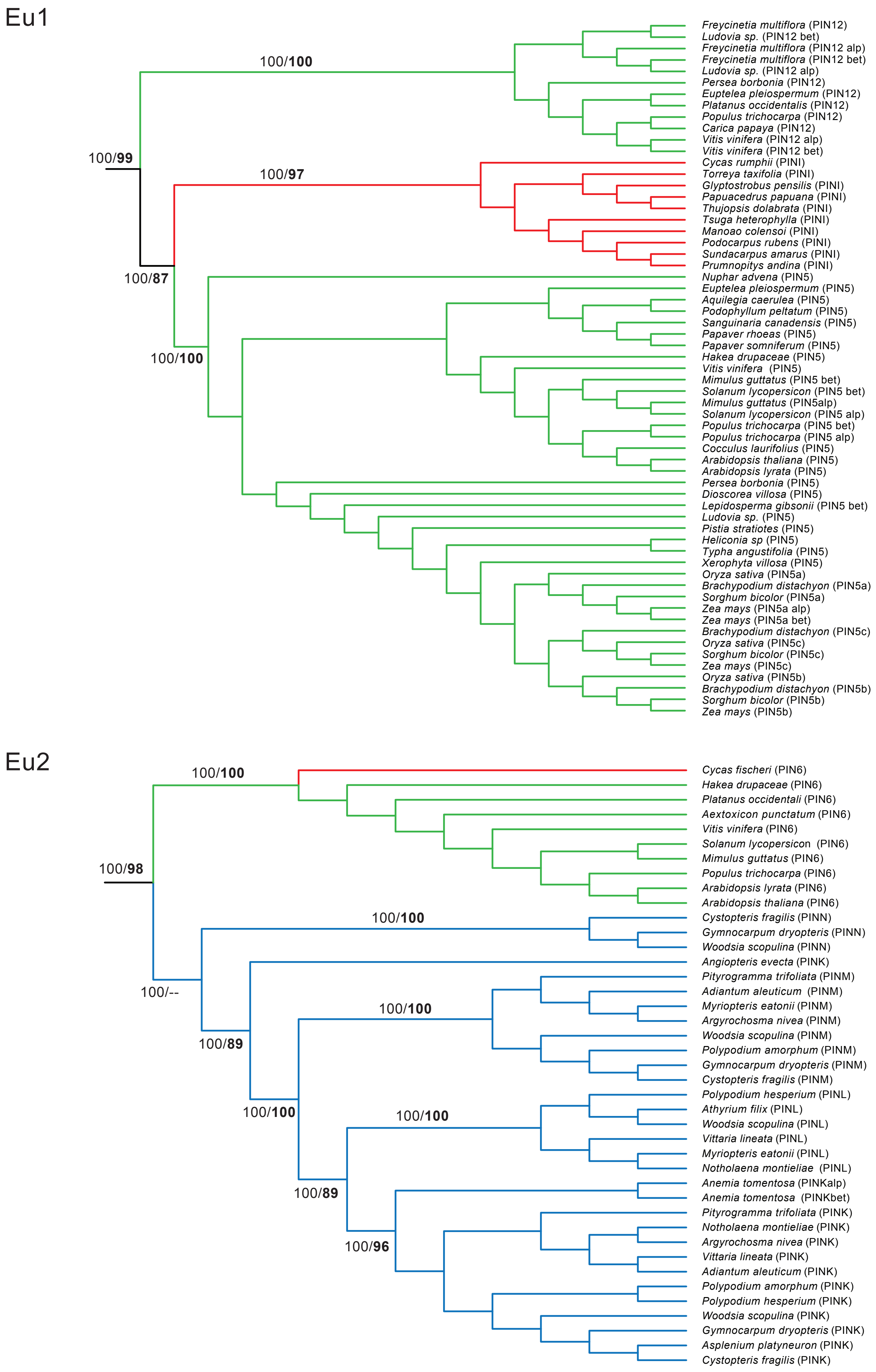
Eu1
100/100
100/99
100/97
100/87
100/100
Freycinetia multiflora (PIN12)
Ludovia sp. (PIN12 bet)
Freycinetia multiflora (PIN12 alp)
Freycinetia multiflora (PIN12 bet)
Ludovia sp. (PIN12 alp)
Persea borbonia (PIN12)
Euptelea pleiospermum (PIN12)
Platanus occidentalis (PIN12)
Populus trichocarpa (PIN12)
Carica papaya (PIN12)
Vitis vinifera (PIN12 alp)
Vitis vinifera (PIN12 bet)
Cycas rumphii (PINI)
Torreya taxifolia (PINI)
Glyptostrobus pensilis (PINI)
Papuacedrus papuana (PINI)
Thujopsis dolabrata (PINI)
Tsuga heterophylla (PINI)
Manoao colensoi (PINI)
Podocarpus rubens (PINI)
Sundacarpus amarus (PINI)
Prumnopitys andina (PINI)
Nuphar advena (PIN5)
Euptelea pleiospermum (PIN5)
Aquilegia caerulea (PIN5)
Podophyllum peltatum (PIN5)
Sanguinaria canadensis (PIN5)
Papaver rhoeas (PIN5)
Papaver somniferum (PIN5)
Hakea drupaceae (PIN5)
Vitis vinifera (PIN5)
Mimulus guttatus (PIN5 bet)
Solanum lycopersicon (PIN5 bet)
Mimulus guttatus (PIN5alp)
Solanum lycopersicon (PIN5 alp)
Populus trichocarpa (PIN5 bet)
Populus trichocarpa (PIN5 alp)
Cocculus laurifolius (PIN5)
Arabidopsis thaliana (PIN5)
Arabidopsis lyrata (PIN5)
Persea borbonia (PIN5)
Dioscorea villosa (PIN5)
Lepidosperma gibsonii (PIN5 bet)
Ludovia sp. (PIN5)
Pistia stratiotes (PIN5)
Heliconia sp (PIN5)
Typha angustifolia (PIN5)
Xerophyta villosa (PIN5)
Oryza sativa (PIN5a)
Brachypodium distachyon (PIN5a)
Sorghum bicolor (PIN5a)
Zea mays (PIN5a alp)
Zea mays (PIN5a bet)
Brachypodium distachyon (PIN5c)
Oryza sativa (PIN5c)
Sorghum bicolor (PIN5c)
Zea mays (PIN5c)
Oryza sativa (PIN5b)
Brachypodium distachyon (PIN5b)
Sorghum bicolor (PIN5b)
Zea mays (PIN5b)
Eu2
100/100
100/98
100/100
100/--
100/89
100/100
100/100
100/100
100/89
100/96
Cycas fischeri (PIN6)
Hakea drupaceae (PIN6)
Platanus occidentali (PIN6)
Aextoxicon punctatum (PIN6)
Vitis vinifera (PIN6)
Solanum lycopersicon (PIN6)
Mimulus guttatus (PIN6)
Populus trichocarpa (PIN6)
Arabidopsis lyrata (PIN6)
Arabidopsis thaliana (PIN6)
Cystopteris fragilis (PINN)
Gymnocarpum dryopteris (PINN)
Woodsia scopulina (PINN)
Angiopteris evecta (PINK)
Pityrogramma trifoliata (PINM)
Adiantum aleuticum (PINM)
Myriopteris eatonii (PINM)
Argyrochosma nivea (PINM)
Woodsia scopulina (PINM)
Polypodium amorphum (PINM)
Gymnocarpum dryopteris (PINM)
Cystopteris fragilis (PINM)
Polypodium hesperium (PINL)
Athyrium filix (PINL)
Woodsia scopulina (PINL)
Vittaria lineata (PINL)
Myriopteris eatonii (PINL)
Notholaena montieliae (PINL)
Anemia tomentosa (PINKalp)
Anemia tomentosa (PINKbet)
Pityrogramma trifoliata (PINK)
Notholaena montieliae (PINK)
Argyrochosma nivea (PINK)
Vittaria lineata (PINK)
Adiantum aleuticum (PINK)
Polypodium amorphum (PINK)
Polypodium hesperium (PINK)
Woodsia scopulina (PINK)
Gymnocarpum dryopteris (PINK)
Asplenium platyneuron (PINK)
Cystopteris fragilis (PINK)
