# Supplementary material for: Paralogous Radiations of PIN Proteins with Multiple Origins of Noncanonical PIN Structure
Source: Mol Biol Evol. 2014 Apr 23;31(8):2042–60. doi: 10.1093/molbev/msu147 (PMC4104312; doi:10.1093/molbev/msu147)
Supplement: Supplementary Data [file supp_msu147_Supplementary_dataset_2.docx]

|  | Helix 1 | Helix 2 | Helix 3 | Helix 4 | Helix 5 | Loop | Helix 6 | Helix 7 | Helix 8 | Helix 9 | Helix 10 |
| --- | --- | --- | --- | --- | --- | --- | --- | --- | --- | --- | --- |
| Refined  modal | 7L -  28V | 39C -  60S | 71F -  93S | 100W -  121L | 132L -  152F |  | 5L -  25F | 39C -  60S | 71V -  93A | 98C -  120A | 130L -  152L |
| AtPIN1 | 10V –  28V | 41G –  61A | 71F –  89W | 96G –  118I | 133M –  152F | 311 | 13S –  35I | 46G –  67C | 73A –  94V | 98A –  120G | 130L –  152L |
|  |  |  |  |  |  |  |  |  | Pred: 89V – 110L | |  |
| AtPIN2 | 10V –  32G | 39C –  61S | 71F –  93S | 100W –  122R | 132L –  154F | 336 | 5L –  28W | 40I –  62Q | 72V –  94A | 101L –  120F | 129I –  151L |
| AtPIN3 | 10V –  29R | 41G –  61T | 71F –  93T | 100W –  122I | 132L –  154F | 329 | 11T –  30A | 45A –  67C | 74F –  96L | 103V –  118V | 129I –  151L |
| AtPIN4 | 7L –  29Q | 44R –  61T | 68N –  90A | 100W –  122I | 129A –  151L | 301 | 5L –  27W | 39I –  61Q | 76M –  98G | 103I –  120A | 132T –  152L |
| AtPIN5 | 10V –  32H | 45L –  62V | 72I –  89W | 96G –  118V | 133V –  152L | 38 | 1V –  23I | 38S –  60L | 73V –  96G | 103V –  120A | 130L –  152L |
| AtPIN6 | 6E –  28V | 41G –  61Q | 71F –  93F | 100W –  122Q | 132L –  152L | 259 | 13S –  31M | 37F –  59A | 74L –  96M | 103A –  120A | 130L –  152L |
| AtPIN7 | 10V –  29R | 42I –  61S | 71F –  93T | 100W –  122I | 132L –  154Y | 308 | 10N –  29V | 38S –  60L | 75A –  96L | 103V –  120A | 130L –  152L |
| AtPIN8 | 7I –  26L | 41G –  61E | 71L –  93W | 103L –  125L | 135A –  157F | 51 | 4L –  23L | 38S –  60S | 72A –  94I | 103V –  120A | 129I –  152L |
| PtPIN1α | 10V -  28V | 40S -  59I | 69F -  91M | 98L -  120L | 130G -  152F | 297 | 5L -  27W | 39I -  61Q | 71V -  93A | 100L -  119F | 129I -  152L |
| PtPIN1β | 10V -  28V | 41G -  60S | 70R -  89W | 96G -  118I | 133M -  152F | 302 | 5L -  29V | 39I -  61Q | 71V -  93A | 98G -  120A | 130L -  152L |
| PtPIN2α | 10V -  28V | 40S -  59I | 69F -  91A | 98L -  120L | 130G -  152F | 321 | 5L -  27W | 34I -  56L | 71V -  93A | 98G -  120A | 130L -  151L |
| PtPIN2β | 10V -  28V | 40A -  59I | 69L -  91A | 98L -  120L | 130S -  152F | 235 | 12Y -  29I | 39V -  61Q | 68G -  90T | 100L -  117A | 130P -  149L |
| PtPIN3α | 10V -  29R | 42I -  61T | 71F -  93T | 100W -  122T | 132T -  154F | 322 | 5L -  29V | 39I -  61Q | 71V -  93A | 98G -  120A | 130L -  152L |
| PtPIN3β | 10V -  29R | 42I -  61T | 71F -  93T | 100W -  122I | 132L -  154F | 337 | 5L -  29V | 39I -  61Q | 71V -  93A | 98G -  120A | 130L -  152L |
| PtPIN5α | 7I -  26G | 41A -  60A | 67M -  89W | 99G -  121I | 133V -  152L | 32 | 5L -  24A | 37G -  59A | 74I -  96L | 103V -  120A | 130L -  152L |
| PtPIN5β | 10V -  28V | 40G -  62V | 72I -  89W | 96G -  118V | 133V -  152L | 32 | 1V -  23I | 38S -  60L | 67C -  89I | 100V -  120A | 130L -  152L |
| PtPIN6β | 10V -  28V | 41G -  61Q | 71F -  93F | 100L -  119L | 129Q -  151F | 223 | 13S -  35V | 45A -  67C | 74M -  94V | 98G -  120A | 130M -  152L |
| PtPIN8α | 7V -  29R | 40S -  62I | 71L -  93S | 100W -  122R | 132L -  154L | 44 | 9P -  28G | 38S -  60L | 72A -  94V | 98G -  120A | 130L -  152L |
| PtPIN8β | 7V -  26I | 40S -  62I | 71L -  93S | 100W -  122R | 132L -  154L | 43 | 1V -  23I | 38S -  60S | 72A -  94V | 98G -  120A | 130L -  152L |
| PtPIN11α | 10V -  28V | 41G -  60S | 71F -  93I | 98E -  120L | 130G -  152F | 275 | 5L -  25F | 29I -  56L | 71V -  93A | 98G -  120A | 130L -  152L |
| PtPIN11β | 7L -  26G | 41G -  60S | 67M -  89W | 98L -  121L | 130G -  152F | 276 | 10N -  35I | 39I -  61Q | 71I -  93A | 100L -  119F | 129I -  151L |
| PtPIN12 | 7L -  26A | 41G -  60S | 70L -  92F | 99E -  121L | 131G -  153E | 58 | 11S -  30M | 45A -  67L | 74Y -  96L | 103L -  120A | 129V -  151F |
| VvPIN1 | 5L -  27S | 40S -  59I | 69F -  91N | 98L -  120L | 130G -  152F | 292 | 6I -  23V | 39I -  61Q | 71I -  93A | 98G -  120A | 130A -  152L |
| VvPIN2 | 7L -  25Y | 40A -  59I | 71F -  93A | 99E -  121L | 128F -  150F | 318 | 5L -  27W | 34I -  56L | 71V -  93A | 98G -  120A | 130I -  152L |
| VvPIN3 | 7L -  26G | 41G -  60S | 71F -  93T | 100W -  122I | 132L -  154Y | 337 | 10N -  29V | 39I -  61Q | 71V -  93A | 98G -  120A | 130L -  152L |
| VvPIN5α | 10V -  32R | 45L -  62V | 67M -  89W | 96G -  118V | 133V -  152L | 42 | 1V -  23I | 38S -  60M | 73V -  95G | 100V -  120A | 130L -  152L |
| VvPIN6 | 10V -  28V | 41G -  61Q | 71F -  93F | 100L -  122A | 132M -  151F | 216 | 13S -  35V | 45A -  67C | 80F -  99V | 103A -  125L | 129I -  151L |
| VvPIN8 | 7V -  26I | 41G -  60S | 71L -  90A | 97S -  119P | 129A -  151L | 45 | 1V -  23I | 38S -  60S | 73V -  95L | 103V -  120A | 129I -  151L |
| VvPIN11α | 7L -  26G | 41G -  60S | 71F -  90S | 98L -  120L | 130G -  152F | 243 | 5L -  27W | 39I -  61Q | 71V -  93A | 98G -  120A | 130I -  152L |
| VvPIN11β | 7L -  26G | 41G -  60S | 71F -  93S | 98L -  120L | 130G -  152F | 243 | 10N -  29I | 38I -  60Q | 70I -  92A | 97G -  119A | 129L -  151L |
| VvPIN12α | 7L -  25Y | 40A -  62I | 69F -  90A | 102I -  121L | 132L -  154Y | 142 | 1V -  23A | 38S -  60L | 72A -  94V | 100V -  120S | 130L -  152L |
| VvPIN12β | 7L -  25Y | 40A -  62I | 69F -  90A | 102I -  121L | 132L -  154Y | 153 | 1V -  23A | 38S -  60L | 72A -  94V | 100V -  120S | 130L -  152L |
| SlPIN1 | 10V -  28V | 41G -  61A | 71F -  93S | 100W -  122K | 132L -  154F | 299 | 6I -  29L | 39I -  61Q | 71V -  93A | 100L -  119F | 129I -  151F |
| SlPIN2 | 10V -  28V | 41G -  60S | 70M -  92S | 99W -  121K | 131L -  153Y | 318 | 8N -  29I | 37G -  59A | 74F -  96L | 98G -  120A | 130L -  152L |
| SlPIN3α | 4W -  26G | 39C -  61M | 71F -  93T | 100W -  122I | 132L -  154Y | 341 | 10N -  29V | 39I -  61Q | 71V -  93A | 98G -  120A | 130L -  152L |
| SlPIN3β | 4W -  26G | 39C -  61M | 71F -  93T | 100W -  122I | 132L -  154F | 301 | 10N -  29V | 39I -  61Q | 71V -  93I | 98G -  120A | 130L -  152L |
| SlPIN5α | 10V -  32H | 45F -  62I | 67L -  89W | 96G -  118V | 133V -  155W | 45 | 12Y -  29F | 39I -  61R | 74Y -  96L | 101L -  120A | 130I -  152L |
| SlPIN5β | 10V -  32H | 45L-  62V | 67M -  89W | 96G -  118I | 128V -  150F | 32 | 8N -  24A | 39I -  58V | 71V -  93L | 98G -  120A | 129I -  151I |
| SlPIN8 | 7V -  26I | 41G -  61G | 71L -  89L | 96G -  118I | 133L -  152F | 45 | 5L -  25F | 39I -  58M | 73I -  95G | 100L -  119F | 129I -  151L |
| SlPIN11α | 10V -  28V | 41G -  60A | 70R -  89W | 96G -  118I | 133M -  152F | 282 | 12Y -  35I | 39I -  61S | 71I -  93A | 98G -  120A | 130I -  152L |
| SlPIN11β | 7L -  26G | 41G -  60A | 71F -  93S | 98L -  120L | 130G -  152F | 274 | 10N -  29I | 39I -  61Q | 71I -  93A | 98G -  120A | 130L -  152L |
| OsPIN1a | 7F -  26G | 41G -  60S | 67M -  89W | 99E -  121L | 128F -  150F | 280 | 5L -  27W | 39I -  61Q | 71V -  93A | 98G -  120A | 130L -  152L |
| OsPIN1b | 7F -  26G | 41G -  60S | 67M -  89W | 99E -  121L | 128F -  150F | 283 | 5L -  27W | 39I -  61Q | 71V -  93A | 98G -  120A | 130L -  152L |
| OsPIN2 | 10V -  32G | 39C -  61T | 71F -  93L | 98L -  120L | 130G -  152F | 310 | 10N -  29I | 37G -  59A | 74F -  96L | 98G -  120A | 130L -  152L |
| OsPIN5a | 10V -  29R | 42V -  61M | 71A -  93M | 98V -  120M | 130Q -  152L | 47 | 11T-  30A | 37G -  58M | 64I -  86A | 101L -  120A | 130L -  152L |
| OsPIN5b | 10V -  28V | 41A -  60A | 80L -  102G | 96G -  118V | 133I -  152F | 41 | 9P -  27W | 38S -  59A | 64I -  86A | 103L -  125L | 133A -  155I |
| OsPIN5c | 10V -  32R | 39C -  61R | 71V -  90A | 96G -  118V | 133V -  152F | 51 | 10N -  30S | 39L -  59G | 64V -  83G | 98G -  120A | 129V -  151V |
| OsPIN8 | 7I –  26L | 40S –  61T | 71L –  93C | 100W –  121L | 132L –  154L | 46 | 6V -  29I | 39I -  60L | 73L -  95G | 99V -  120A | 129I -  154I |
| OsPIN9 | 10V -  28V | 41G -  60S | 70R -  92W | 96G -  118I | 133M -  152F | 101 | 4L -  23A | 38L -  57I | 70I -  92V | 97G -  119A | 132G -  151L |
| OsPIN10a | 7S –  26G | 41G –  60S | 67M –  89W | 109L –  131L | 146G –  163Y | 303 | 10N –  31M | 45A –  67C | 72A –  96L | 103V –  120A | 130L –  152L |
|  |  |  |  |  |  |  |  |  | Pred: 88A - 110L | |  |
| OsPIN10b | 10V -  32G | 39C -  61S | 71F -  93P | 106C -  128V | 134L -  156F | 272 | 12Y -  34I | 38S -  57F | 64I -  83G | 98G -  120A | 129I -  151I |
| OsPIN11α | 10V -  32R | 45F -  67M | 71F -  93S | 100W -  121L | 132L -  154Y | 238 | 5L -  29I | 39I -  61Q | 71L -  93A | 98G -  120A | 130L -  152L |
| OsPIN11β | 10V -  32R | 45F -  67M | 71F -  93S | 100W -  121L | 132L -  154Y | 239 | 5L -  29I | 39I -  61Q | 71L -  93A | 98G -  120A | 130L -  152L |
| SbPIN1a | 10V -  29R | 42I -  61T | 71F -  93S | 98L -  120L | 130G -  152F | 293 | 10N -  29F | 39I -  61Q | 71V -  93A | 100L -  119F | 129I -  151L |
| SbPIN1b | 7F -  26G | 41G -  60S | 67M -  89W | 99E -  121L | 128F -  150F | 294 | 5L -  27W | 39I -  61Q | 71V -  93A | 98G -  120A | 130L -  152L |
| SbPIN2 | 10V -  32G | 45F -  67M | 77L -  94S | 100W -  122R | 132L -  154Y | 307 | 10N -  29I | 37G -  59A | 74F -  96L | 98G -  120A | 130L -  152L |
| SbPIN5b | 10V -  32R | 39C -  56F | 71V -  93C | 92A -  115A | 124I -  143F | 86 | 11V -  33I | 33I -  56F | 71A -  93F | 100L -  119A | 129L -  151L |
| SbPIN5c | 10K -  29V | 42A -  61S | 81L -  103G | 106R -  128V | 143I -  162L | 41 | 11V -  30I | 40I -  62F | 75A -  97F | 102L -  121A | 134L -  156L |
| SbPIN8 | 15V –  34F | 41G –  61T | 71L –  89I | 100W –  122K | 132L –  154L | 47 | 5L -  23I | 38S -  60L | 73L -  95G | 98G -  120A | 129I -  151V |
| SbPIN9 | 5S –  27S | 39C –  61T | 71L –  93S | 100W –  121L | 136I –  155M | 118 | 5L -  24A | 39L -  58I | 71I -  93A | 98G -  120A | 133G -  152L |
| SbPIN10b | 10V -  32G | 45F -  67M | 71F -  93P | 110W -  132R | 142L -  164F | 269 | 12Y -  29V | 39I -  58M | 71V -  93A | 98G -  120G | 129I -  151I |
| SbPIN11 | 10V -  28V | 41G -  60S | 80V -  102G | 104L -  126G | 133M -  152F | 258 | 5L -  29I | 39I -  61Q | 71F -  93A | 98G -  120A | 130L -  152L |
| BdPIN1a | 10V -  28V | 42I -  61T | 71F -  93S | 98L -  120L | 130G -  152F | 284 | 12Y -  29F | 39I -  61Q | 74F -  96L | 100L -  120A | 129I -  151L |
| BdPIN1b | 7F -  26G | 41G -  60S | 67M -  89W | 99E -  121L | 128F -  150F | 268 | 5L -  27W | 39I -  61Q | 71V -  93A | 98G -  120A | 130L -  152L |
| BdPIN2 | 10V -  32G | 39C -  61T | 71F -  93L | 98L -  120L | 130G -  152F | 325 | 8N -  27W | 37G -  59A | 74F -  96L | 98G -  120A | 130L -  152L |
| BdPIN5a | 10V -  29R | 42V -  61M | 71A -  93M | 98G -  120V | 135V -  154L | 50 | 5L -  27L | 37G -  59A | 74L –  96L | 103V –  120A | 130L –  152L |
| BdPIN5b | 10V -  28V | 41A -  56F | 71V -  93V | 94L -  116V | 131D -  153E | 41 | 10N -  29I | 37G -  59A | 74L –  96L | 103L –  120A | 130L –  152L |
| BdPIN5c | 7I -  26G | 41A -  63V | 70F -  92W | 102P -  124A | 133L -  155L | 56 | 5V -  27W | 34V -  56L | 69A –  91A | 98G -  120A | 130L -  152L |
| BdPIN8 | 5I –  27S | 39C -  61T | 71L -  93C | 100W -  122K | 132L -  154F | 47 | 11M –  30Q | 45G –  67C | 74L –  96M | 103V –  120A | 129I –  151V |
| BdPIN9 | 10V -  32G | 39C –  61T | 71L –  93P | 94V –  116L | 136I –  158R | 87 | 5V -  24A | 39L -  58I | 71V -  93A | 98G -  120A | 133G -  152L |
| BdPIN10a | 10V -  32G | 39C -  61A | 71F -  93I | 100W -  122V | 132L -  154F | 275 | 11T –  30S | 45A –  67C | 74V –  96L | 103I –  120A | 133A –  155V |
| BdPIN11 | 10V -  28V | 41G -  60S | 80L -  99F | 100W -  122R | 132L -  154Y | 234 | 10N -  29I | 39I -  61Q | 71L -  93A | 98G -  120A | 130L -  152L |
| ZmPIN1a-α | 7F –  26S | 41G -  60S | 71F -  93S | 96G -  118I | 133M -  152F | 283 | 11T –  30Q | 45A –  67C | 74A -  94L | 98G -  120A | 130L -  152L |
|  |  |  |  |  |  |  |  |  | Pred: 89V – 110L | |  |
| ZmPIN1a-β | 7F –  26S | 41G -  60S | 71F -  93S | 100W -  122R | 132L -  154Y | 281 | 10N -  29F | 34I -  56L | 71V -  93A | 98G -  120A | 130L -  152L |
| ZmPIN1b | 10V -  29R | 42I -  61T | 71F -  93S | 98L -  120L | 130G -  152F | 289 | 10N -  29F | 39I -  61Q | 71V -  93A | 100L -  119F | 129I -  151L |
| ZmPIN5a-α | 10V -  28V | 41A -  58F | 73A -  95G | 96G -  118L | 133V -  152L | 62 | 11T -  29I | 37G -  59A | 74L -  96L | 103V -  120A | 130L -  152L |
| ZmPIN5b | 10V -  28V | 41A -  60A | 80L -  97S | 96R -  118V | 133I -  152L | 36 | 10N -  29I | 39V -  60L | 64V -  86A | 101L -  120A | 133A -  155I |
| ZmPIN8 | 15V -  34F | 41G -  61T | 71L -  93C | 100W -  122K | 132L -  154F | 48 | 12Y -  31L | 41I -  60L | 73L -  95G | 100L -  120A | 129I -  151V |
| ZmPIN9 | 5S -  27S | 40A –  59V | 74A –  96S | 117W –  138L | 153I –  172M | 105 | 5L -  24A | 39L -  58I | 71V -  93A | 98G -  120A | 133G -  152L |
| ZmPIN10a | 7V –  26G | 41G -  66Y | 71F -  93P | 100W -  122I | 133L -  154F | 292 | 10N -  29I | 39I -  61Q | 76M –  96L | 103I –  120A | 130L –  152L |
| ZmPIN10b | 7L –  29Q | 44R -  60S | 71F -  93P | 100W -  122V | 132L -  154F | 258 | 13A –  35V | 45A –  67C | 71V -  93A | 98G -  120A | 129I -  151V |
|  |  |  |  |  |  |  |  |  | Pred: 79R – 101L | |  |
| ZmPIN11 | 7L –  26G | 41G -  60S | 71F -  93L | 96L -  118I | 132L -  154Y | 264 | 10N -  29I | 39I -  61Q | 71L -  93A | 98G -  120A | 130L -  152L |
| SmPINR | 10V –  32G | 39C –  61S | 71F –  93S | 100W –  122A | 132L –  153Y | 285 | 12Y –  29I | 39I –  61L | 73A –  95C | 104V –  121A | 130I –  152L |
| SmPINS | 4A –  26G | 39C –  61T | 71F –  93S | 100W –  122A | 132L –  153Y | 313 | 12Y –  34I | 39S –  60L | 72A –  94V | 101L –  120A | 129V –  151L |
| SmPINT | 10V –  32G | 45F –  67M | 71F –  93S | 100W –  122Y | 132L –  153Y | 290 | 11T –  29Y | 38S –  60L | 75G –  96L | 103A –  120A | 130L –  152L |
| SmPINUα | 10V –  32G | 39C –  61H | 71F –  93S | 100W –  122N | 132L –  153Y | 270 | 11T –  29L | 38G –  59A | 74I –  96L | 103A –  120A | 130L –  152L |
| SmPINUβ | 10V –  32G | 39C –  61H | 71F –  93S | 100W –  122N | 132L –  153Y | 214 | 11T –  29L | 35I –  57F | 72T –  94A | 101L –  118A | 128L –  150L |
| SmPINV | 10V –  28V | 41G –  60A | 70R –  92L | 112W –  134V | 144L –  166Y | 300 | 8N –  29V | 45A –  67C | 72A –  94V | 103A –  120A | 129V –  151L |
| PpPINA | 10V –  32G | 39C –  61G | 71F –  93A | 100W –  122A | 132L –  153Y | 402 | 5L –  24A | 40H –  61G | 74F –  95G | 103V –  120A | 129I –  151L |
| PpPINB | 10V -  32G | 39C –  61G | 71F –  93S | 100W –  122A | 132L –  153Y | 401 | 5L –  24A | 39V –  61G | 74F –  96L | 103V –  120A | 130L –  152L |
| PpPINC | 10V -  32G | 44R –  61G | 71F –  93S | 100W –  122A | 132L –  153Y | 386 | 12Y –  32I | 39Y –  61G | 73L –  95G | 103V –  120A | 131S –  152L |
| PpPIND | 10V -  32G | 39S –  61F | 71L –  93T | 100W –  122S | 132I –  150Y | 120 | 6V –  28G | 41I –  60G | 65V –  87G | 102V –  119A | 132A –  151L |
