# Supplementary material for: Paralogous Radiations of PIN Proteins with Multiple Origins of Noncanonical PIN Structure
Source: Mol Biol Evol. 2014 Apr 23;31(8):2042–60. doi: 10.1093/molbev/msu147 (PMC4104312; doi:10.1093/molbev/msu147)
Supplement: Supplementary Data [file supp_msu147_Supplementary_dataset_3.docx]

| **Species** | **Accession number** | | **Origin** | **Old name** | **New name** |
| --- | --- | --- | --- | --- | --- |
|  |  | |  |  |  |
| **Marchantiophyta** |  | |  |  |  |
| *Marchantia polymorpha* | | isotig18884 | Marchantia Genome Project |  | MarpoPINW |
| *Marchantia polymorpha* | | isotig26031, c2201423 | Marchantia Genome Project |  | MarpoPINX |
| *Marchantia polymorpha* | | isotig29057, c2201504 | Marchantia Genome Project |  | MarpoPINY |
| *Marchantia polymorpha* | JPYU-0056667 | | 1KP project |  | MarpoPINZ |
|  |  | |  |  |  |
| **Bryophyta** |  | |  |  |  |
| *Physcomitrella patens* | Pp1s10_17V6.1 | | Cosmoss | PpPINA | PhypaPINA |
| *Physcomitrella patens* | Pp1s18_186V6.1 | | Cosmoss | PpPINB | PhypaPINB |
| *Physcomitrella patens* | Pp1s32_43V6.1 | | Cosmoss | PpPINC | PhypaPINC |
| *Physcomitrella patens* | Pp1s79_126V6.1 | | Cosmoss | PpPIND | PhypaPINDα |
| *Physcomitrella patens* | Pp1s402_39V6.1 | | Cosmoss |  | PhypaPINDβ |
| *Polytrichum commume* | SZYG-0018815 | | 1KP project |  | PolcoPINA |
| *Sphagnum lescurii* | GOWD-1209084 | | 1KP project |  | SphlePINAα |
| *Sphagnum lescurii* | GOWD-1035686 | | 1KP project |  | SphlePINAβ |
|  |  | |  |  |  |
| **Lycophyta** |  | |  |  |  |
| **Isoetopsida** |  | |  |  |  |
| *Selaginella moellendorfii* | Selmo1-99301 | | Phytozome |  | SelmoPINR |
| *Selaginella moellendorfii* | Selmo1-234325 | | Phytozome |  | SelmoPINS |
| *Selaginella moellendorfii* | Selmo1-102666 | | Phytozome |  | SelmoPINT |
| *Selaginella moellendorfii* | Selmo1-268490 | | Phytozome |  | SelmoPINUα |
| *Selaginella moellendorfii* | Selmo1-231064 | | Phytozome |  | SelmoPINUβ |
| *Selaginella moellendorfii* | Selmo1-119024 | | Phytozome |  | SelmoPINV |
| *Selaginella apoda* | LGDQ-0009330 + LGDQ-0012407 | | 1KP project |  | SelapPINR |
| *Selaginella apoda* | LGDQ-0069396 + LGDQ-0047671 | | 1KP project |  | SelapPINS |
| *Selaginella apoda* | LGDQ-0015988 | | 1KP project |  | SelapPINT |
| *Selaginella apoda* | LGDQ-0009898 | | 1KP project |  | SelapPINV |
|  |  | |  |  |  |
| **Lycopodiopsida** |  | |  |  |  |
| *Huperzia squarrosa* | GAON-0062265 | | 1KP project |  | HupsqPINOα |
| *Huperzia squarrosa* | GAON-0051651 | | 1KP project |  | HupsqPINOβ |
| *Huperzia squarrosa* | GAON-0039862 | | 1KP project |  | HupsqPINOγ |
| *Huperzia squarrosa* | GAON-0012509 | | 1KP project |  | HupsqPINOδ |
| *Huperzia squarrosa* | GAON-0014553 | | 1KP project |  | HupsqPINP |
| *Huperzia squarrosa* | GAON-0015692 | | 1KP project |  | HupsqPINQα |
| *Huperzia squarrosa* | GAON-0009602 | | 1KP project |  | HupsqPINQβ |
|  |  | |  |  |  |
| **Monilophytes** |  | |  |  |  |
| **Equisetopsida** |  | |  |  |  |
| *Equisetum diffusum* | CAPN-1049228 | | 1KP project |  | EqudiPINK |
| *Equisetum diffusum* | CAPN-1059593 | | 1KP project |  | EqudiPINJ |
|  |  | |  |  |  |
| **Psilotopsida** |  | |  |  |  |
| *Psilotum nudum* | QVMR-0023092 | | 1KP project |  | PsinuPINNα |
| *Psilotum nudum* | QVMR-0000213 | | 1KP project |  | PsinuPINNβ |
| *Psilotum nudum* | QVMR-0001666 | | 1KP project |  | PsinuPINJ |
|  |  | |  |  |  |
| **Marritiopsida** |  | |  |  |  |
| *Angiopteris evecta* | NHCM-0024117 | | 1KP project |  | AngevPINK |
| *Angiopteris evecta* | NHCM-0002287 | | 1KP project |  | AngevPINM |
|  |  | |  |  |  |
| **Polypodiopsdia** |  | |  |  |  |
| *Anemia tomentosa* | CQPW-0153856 | | 1KP project |  | AnetoPINKα |
| *Anemia tomentosa* | CQPW-0151291 | | 1KP project |  | AnetoPINKβ |
| *Azolla caroliniana* | CVEG-0130273 | | 1KP project |  | AzocaPINL |
| *Azolla caroliniana* | CVEG-0036202 | | 1KP project |  | AzocaPINJ |
| *Pityrogramma trifoliata* | UJTT-0125912 | | 1KP project |  | PittrPINK |
| *Pityrogramma trifoliata* | UJTT-0116135 | | 1KP project |  | PittrPINLα |
| *Pityrogramma trifoliata* | UJTT-0022723 | | 1KP project |  | PittrPINLβ |
| *Pityrogramma trifoliata* | UJTT-0119528 | | 1KP project |  | PittrPINM |
| *Pityrogramma trifoliata* | UJTT-0014929 | | 1KP project |  | PittrPINN |
| *Myriopteris eatonii* | GSXD-0098472 | | 1KP project |  | MyreaPINL |
| *Myriopteris eatonii* | GSXD-0025780 | | 1KP project |  | MyreaPINM |
| *Myriopteris eatonii* | GSXD-0022480 | | 1KP project |  | MyreaPINN |
| *Argyrochosma nivea* | XDDT-0076068 + XDDT-0112420 | | 1KP project |  | ArgniPINK |
| *Argyrochosma nivea* | XDDT-0098766 | | 1KP project |  | ArgniPINL |
| *Argyrochosma nivea* | XDDT-0002423 | | 1KP project |  | ArgniPINM |
| *Argyrochosma nivea* | XDDT-0109037 | | 1KP project |  | ArgniPINN |
| *Notholaena montieliae* | YCKE-0084087 | | 1KP project |  | NotmoPINK |
| *Notholaena montieliae* | YCKE-0079356 | | 1KP project |  | NotmoPINL |
| *Notholaena montieliae* | YCKE-0024475 | | 1KP project |  | NotmoPINM |
| *Vittaria lineata* | SKYV-0007766 | | 1KP project |  | VitliPINK |
| *Vittaria lineata* | SKYV-0024833 | | 1KP project |  | VitliPINL |
| *Vittaria lineata* | SKYV-0014396 | | 1KP project |  | VitliPINN |
| *Vittaria lineata* | SKYV-0021381 | | 1KP project |  | VitliPINJ |
| *Adiantim aleuticum* | WCLG-0005677 | | 1KP project |  | AdiαINK |
| *Adiantim aleuticum* | WCLG-0013269 | | 1KP project |  | AdiαINM |
| *Adiantim aleuticum* | WCLG-0000010 | | 1KP project |  | AdiαINN |
| *Adiantim aleuticum* | WCLG-0001091 | | 1KP project |  | AdiαINJ |
| *Gymnocarpum dryopteris* | HEGQ-0001833 + HEGQ-0008768 | | 1KP project |  | GymdrPINK |
| *Gymnocarpum dryopteris* | HEGQ-0103396 + HEGQ-0025455 | | 1KP project |  | GymdrPINM |
| *Gymnocarpum dryopteris* | HEGQ-0025694 | | 1KP project |  | GymdrPINN |
| *Cystopteris fragilis* | LHLE-0112105 | | 1KP project |  | CysfrPINK |
| *Cystopteris fragilis* | LHLE-0099063 | | 1KP project |  | CysfrPINL |
| *Cystopteris fragilis* | LHLE-0099397 + LHLE-0113160 | | 1KP project |  | CysfrPINM |
| *Cystopteris fragilis* | LHLE-0113043 | | 1KP project |  | CysfrPINN |
| *Cystopteris fragilis* | LHLE-0096079 | | 1KP project |  | CysfrPINJ |
| *Asplenium platyneuron* | KJZG-0003535 | | 1KP project |  | AspplPINK |
| *Asplenium platyneuron* | KJZG-0000996 | | 1KP project |  | AspplPINL |
| *Woodsia scopulina* | YJJY-0108969 | | 1KP project |  | WooscPINK |
| *Woodsia scopulina* | YJJY-0110963 | | 1KP project |  | WooscPINL |
| *Woodsia scopulina* | YJJY-0004392 | | 1KP project |  | WooscPINM |
| *Woodsia scopulina* | YJJY-0028189 | | 1KP project |  | WooscPINN |
| *Athyrium filix* | URCP-0075276 | | 1KP project |  | AthfiPINK |
| *Athyrium filix* | URCP-0007110 | | 1KP project |  | AthfiPINL |
| *Athyrium filix* | URCP-0075276 | | 1KP project |  | AthfiPINN |
| *Polypodium hesperium* | GYFU-0033216 | | 1KP project |  | PolhePINJ |
| *Polypodium hesperium* | GYFU-0001887 | | 1KP project |  | PolhePINK |
| *Polypodium hesperium* | GYFU-0165910 | | 1KP project |  | PolhePINL |
| *Polypodium amorphum* | YLJA-0012667 | | 1KP project |  | PolamPINK |
| *Polypodium amorphum* | YLJA-0084501 | | 1KP project |  | PolamPINL |
| *Polypodium amorphum* | YLJA-0107939 | | 1KP project |  | PolamPINM |
| *Polypodium amorphum* | YLJA-0097977 + YLJA-0092707 | | 1KP project |  | PolamPINJ |
|  |  | |  |  |  |
| **Gnetophyta** |  | |  |  |  |
| *Welwitschia mirablis* | DT592707 | | TIGR ESTs |  | WelmiPINE |
| *Welwitschia mirablis* | DT592706 | | TIGR ESTs |  | WelmiPING |
|  |  | |  |  |  |
| **Cycadophyta** |  | |  |  |  |
| *Cycas rumphii* | CB091749 | | TIGR ESTS |  | CycruPINI |
| *Zamia fischerii* | DY036528 | | TIGR ESTS |  | ZamfiPIN6K |
| *Zamia fischeri* | 0009221 | | 1KP project |  | ZamfiPINH |
| *Zamia fischeri* | 0022257 | | 1KP project |  | ZamfiPINI |
|  |  | |  |  |  |
| **Pinophyta** |  | |  |  |  |
| **Pinaceae** |  | |  |  |  |
| *Picea glauca* | TC145001, DR557272, DR589633, TA24930_3330, | | TGI ESTs, TIGR ESTs |  | PicglPINE |
| *Picea glauca* | TC150645, TA20938_3330 | | TGI ESTs, TIGR ESTs |  | PicglPING |
| *Picea glauca* | TC157054, CO480051 | | TGI ESTs, TIGR ESTs |  | PicglPINH |
| *Pinus taeda* | TC149669, TC194236, TA11958_3352, NXNV_129_F06 | | TGI ESTs, TIGR ESTs |  | PintaPINE |
| *Pinus taeda* | TC136420, TC179604 | | TGI ESTs |  | PintaPING |
| *Picea abies* | Genbank: ACH91613.2 | | Genbank |  | PicabPINE |
| *Cedrus libani* | GGEA-0012859 | | 1KP project |  | CedliPINE |
| *Cedrus libani* | GGEA-0010339 | | 1KP project |  | CedliPINH |
| *Cedrus libani* | GGEA-0062792 | | 1KP project |  | CedliPINI |
| *Tsuga heterophylla* | GAMH-0097976 | | 1KP project |  | TsuhePING |
| *Tsuga heterophylla* | GAMH-0079417 | | 1KP project |  | TsuhePINH |
| *Tsuga heterophylla* | GAMH-0090160 | | 1KP project |  | TsuhePINI |
| *Pseudotsuga menziesii* | IOVS-0062527 | | 1KP project |  | PsemePINE |
| *Pseudotsuga menziesii* | IOVS-0005480 | | 1KP project |  | PsemePINH |
| *Keteleeria evelyniana* | JUWL-0130560 | | 1KP project |  | KetevPINE |
| *Keteleeria evelyniana* | JUWL-0122586 | | 1KP project |  | KetevPING |
| *Keteleeria evelyniana* | JUWL-0097922 | | 1KP project |  | KetevPINH |
|  |  | |  |  |  |
| **Podocarpaceae** |  | |  |  |  |
| *Podocarpus coriaceus* | SCEB-0078003 | | 1KP project |  | PodcoPINE |
| *Podocarpus coriaceus* | SCEB-0020633 | | 1KP project |  | PodcoPINF |
| *Podocarpus coriaceus* | SCEB-0019164 | | 1KP project |  | PodcoPING |
| *Podocarpus rubens* | XLGK-0009678 | | 1KP project |  | PodruPINF |
| *Podocarpus rubens* | XLGK-0020477 | | 1KP project |  | PodruPING |
| *Podocarpus rubens* | XLGK-0004952 | | 1KP project |  | PodruPINH |
| *Podocarpus rubens* | XLGK-0020828 | | 1KP project |  | PodruPINI |
| *Nageia nagi* | UUJS-0214257 | | 1KP project |  | NagnaPINE |
| *Nageia nagi* | UUJS-0200469 + UUJS-0212581 | | 1KP project |  | NagnaPINF |
| *Nageia nagi* | UUJS-0211682 | | 1KP project |  | NagnaPINI |
| *Sundacarpus amarus* | KLGF-0117782 | | 1KP project |  | SunamPINE |
| *Sundacarpus amarus* | KLGF-0113888 + KLGF-0141487 | | 1KP project |  | SunamPING |
| *Sundacarpus amarus* | KLGF-0143301 | | 1KP project |  | SunamPINI |
| *Dacrycarpus compactus* | FMWZ-0089190 | | 1KP project |  | DaccoPINF |
| *Dacrycarpus compactus* | FMWZ-0020625 | | 1KP project |  | DaccoPING |
| *Dacrycarpus compactus* | FMWZ-0015750 | | 1KP project |  | DaccoPINH |
| *Prumnopitys andina* | EGLZ-0016529 | | 1KP project |  | PruanPINE |
| *Prumnopitys andina* | EGLZ-0014349 | | 1KP project |  | PruanPINF |
| *Prumnopitys andina* | EGLZ-0012361 | | 1KP project |  | PruanPING |
| *Prumnopitys andina* | EGLZ-0007480 | | 1KP project |  | PruanPINH |
| *Prumnopitys andina* | EGLZ-0009600 | | 1KP project |  | PruanPINI |
| *Manoao colensoi* | CDFR-0003524 | | 1KP project |  | MancoPINE |
| *Manoao colensoi* | CDFR-0113347 | | 1KP project |  | MancoPINF |
| *Manoao colensoi* | CDFR-0010808 | | 1KP project |  | MancoPINH |
| *Manoao colensoi* | CDFR-0127419 | | 1KP project |  | MancoPINI |
|  |  | |  |  |  |
| **Sciadopityaceae** |  | |  |  |  |
| *Sciadopitys verticillata* | YFZK-0011791 | | 1KP project |  | ScivePINE |
| *Sciadopitys verticillata* | YFZK-0019075 | | 1KP project |  | ScivePINF |
| *Sciadopitys verticillata* | YFZK-0011623 | | 1KP project |  | ScivePINGa |
|  |  | |  |  |  |
| **Cupressaceae** |  | |  |  |  |
| *Cryptomeria japonica* | BY879231.1, BY888584.1, BY895451.1 | | Genbank |  | CryjaPINE |
| *Chamaecyparis lawsoniana* | AIGO-0133123 | | 1KP project |  | ChalaPINE |
| *Chamaecyparis lawsoniana* | AIGO-0009705 | | 1KP project |  | ChalaPINF |
| *Chamaecyparis lawsoniana* | AIGO-0006964 + AIGO-0129693 | | 1KP project |  | ChalaPINGa |
| *Chamaecyparis lawsoniana* | AIGO-0009365 | | 1KP project |  | ChalaPINGb |
| *Chamaecyparis lawsoniana* | AIGO-0117511 | | 1KP project |  | ChalaPINH |
| *Chamaecyparis lawsoniana* | AIGO-0095784 | | 1KP project |  | ChalaPINI |
| *Juniperus scopulorum* | XMGP-0020781 | | 1KP project |  | JunscPINGa |
| *Juniperus scopulorum* | XMGP-0017386 | | 1KP project |  | JunscPINGb |
| *Thuja plicata* | VFYZ-0017779 | | 1KP project |  | ThuplPINE |
| *Thuja plicata* | VFYZ-0014539 | | 1KP project |  | ThuplPINF |
| *Thuja plicata* | VFYZ-0009562 | | 1KP project |  | ThuplPINGa |
| *Thuja plicata* | VFYZ-0009412 | | 1KP project |  | ThuplPINH |
| *Thujopsis dolabrata* | NKIN-0014548 | | 1KP project |  | ThudoPINF |
| *Thujopsis dolabrata* | NKIN-0004948 | | 1KP project |  | ThudoPINGa |
| *Thujopsis dolabrata* | NKIN-0004954 | | 1KP project |  | ThudoPINH |
| *Thujopsis dolabrata* | NKIN-0016320 | | 1KP project |  | ThudoPINI |
| *Fokienia hodginsii* | UEVI-0020408 | | 1KP project |  | FokhoPINE |
| *Fokienia hodginsii* | UEVI-0093976 | | 1KP project |  | FokhoPINF |
| *Fokienia hodginsii* | UEVI-0073350 | | 1KP project |  | FokhoPINGa |
| *Glyptostrobus pensilis* | OXGJ-0011964 | | 1KP project |  | GlypePINE |
| *Glyptostrobus pensilis* | OXGJ-0017350 | | 1KP project |  | GlypePINF |
| *Glyptostrobus pensilis* | OXGJ-0017006 | | 1KP project |  | GlypePINGa |
| *Glyptostrobus pensilis* | OXGJ-0014196 | | 1KP project |  | GlypePINGb |
| *Glyptostrobus pensilis* | OXGJ-0002800 | | 1KP project |  | GlypePINI |
| *Papuacedrus papuana* | OVIJ-0010850 | | 1KP project |  | PappaPINE |
| *Papuacedrus papuana* | OVIJ-0114928 | | 1KP project |  | PappaPINF |
| *Papuacedrus papuana* | OVIJ-0030377 + OVIJ-0098754 | | 1KP project |  | PappaPINGa |
| *Papuacedrus papuana* | OVIJ-0015704 + OVIJ-0107199 | | 1KP project |  | PappaPINH |
| *Papuacedrus papuana* | OVIJ-0113736 | | 1KP project |  | PappaPINI |
| *Cunninghamia lanceolata* | OUOI-0066642 | | 1KP project |  | CunlaPINE |
| *Cunninghamia lanceolata* | OUOI-0060475 | | 1KP project |  | CunlaPINGa |
|  |  | |  |  |  |
| **Cephalotaxaceae** |  | |  |  |  |
| *Torreya taxifolia* | EFMS-0083638 + EFMS-0111819 + EFMS-0103430 | | 1KP project |  | TortaPINF |
| *Torreya taxifolia* | EFMS-0029883 | | 1KP project |  | TortaPINGa |
| *Torreya taxifolia* | EFMS-0088071 + EFMS-0109113 | | 1KP project |  | TortaPINGb |
| *Torreya taxifolia* | EFMS-0012912 | | 1KP project |  | TortaPINI |
| *Torreya nucifera* | HQOM-0133659 | | 1KP project |  | TornuPINF |
| *Amentotaxus argotaenia* | IAJW-0063419 | | 1KP project |  | AmearPINE |
| *Amentotaxus argotaenia* | IAJW-0062552 | | 1KP project |  | AmearPINF |
| *Amentotaxus argotaenia* | IAJW-0057505 | | 1KP project |  | AmearPINGa |
| *Amentotaxus argotaenia* | IAJW-0003742 + IAJW-0185552 | | 1KP project |  | AmearPINGb |
| *Amentotaxus argotaenia* | IAJW-0014455 | | 1KP project |  | AmearPINH |
|  |  | |  |  |  |
| **Taxaceae** |  | |  |  |  |
| *Taxus baccata* | WWSS-0053311 | | 1KP project |  | TaxbaPINE |
| *Taxus baccata* | WWSS-0051542 + WWSS-0016587 | | 1KP project |  | TaxbaPINF |
| *Taxus baccata* | WWSS-0014183 | | 1KP project |  | TaxbaPINGa |
| *Taxus baccata* | WWSS-0037063 | | 1KP project |  | TaxbaPINI |
|  |  | |  |  |  |
| **Angiosperms** |  | |  |  |  |
| **Amborellales** |  | |  |  |  |
| *Amborella trichopoda* | URDJ-0098928 + URDJ-0002535 | | 1KP project |  | AmbtrPIN1 |
| *Amborella trichopoda* | URDJ-0228412 + URDJ-0024156 + URDJ-0005341 | | 1KP project |  | AmbtrPIN3 |
| *Amborella trichopoda* | URDJ-0082976 | | 1KP project |  | AmbtrPIN11 |
| *Amborella trichopoda* | URDJ-0119349 | | 1KP project |  | AmbtrPIN12 |
|  |  | |  |  |  |
| **Nympheales** |  | |  |  |  |
| *Nuphar advena* | WTKZ-0011937 | | 1KP project |  | NupadPIN1α |
| *Nuphar advena* | WTKZ-0023751 | | 1KP project |  | NupadPIN1β |
| *Nuphar advena* | WTKZ-0030629 | | 1KP project |  | NupadPIN2α |
| *Nuphar advena* | WTKZ-0059977 + ?WTKZ-0089969 | | 1KP project |  | NupadPIN2β |
| *Nuphar advena* | WTKZ-0058179 + WTKZ-0063771 | | 1KP project |  | NupadPIN3α |
| *Nuphar advena* | WTKZ-0060522 + WTKZ-0074594 | | 1KP project |  | NupadPIN3β |
| *Nuphar advena* | WTKZ-0058179 | | 1KP project |  | NupadPIN3γ |
| *Nuphar advena* | WTKZ-0088382 | | 1KP project |  | NupadPIN5 |
| *Nuphar advena* | WTKZ-0045017 | | 1KP project |  | NupadPIN11α |
| *Nuphar advena* | WTKZ-0034893 + WTKZ-0009125 | | 1KP project |  | NupadPIN11β |
| *Nuphar advena* | WTKZ-0053021 + WTKZ-0009301 + ?WTKZ-0097503 | | 1KP project |  | NupadPIN11γ |
| *Nuphar advena* | WTKZ-0082032 | | 1KP project |  | NupadPIN11δ |
| *Nuphar advena* | WTKZ-0091411 | | 1KP project |  | NupadPIN12 |
|  |  | |  |  |  |
| **Magnoliales** |  | |  |  |  |
| *Liriodendron tulipifera* | 0006887 | | AAGP |  | LirtuPIN1α |
| *Liriodendron tulipifera* | 0008493 | | AAGP |  | LirtuPIN1β |
| *Liriodendron tulipifera* | 0037370 | | AAGP |  | LirtuPIN2 |
| *Liriodendron tulipifera* | 0057034 | | AAGP |  | LirtuPIN6 |
| *Liriodendron tulipifera* | 0005510 | | AAGP |  | LirtuPIN3 |
| *Liriodendron tulipifera* | 0006181 | | AAGP |  | LirtuPIN11 |
| *Liriodendron tulipifera* | 0016254 | | AAGP |  | LirtuPIN12 |
|  |  | |  |  |  |
| **Piperales** |  | |  |  |  |
| *Aristolochia elegans* | lrc14465 | | AAGP |  | ArielPIN1 |
| *Aristolochia elegans* | 0122752 | | AAGP |  | ArielPIN2 |
| *Aristolochia elegans* | 0040644 | | AAGP |  | ArielPIN3 |
| *Aristolochia elegans* | 0045668 | | AAGP |  | ArielPIN8 |
| *Aristolochia elegans* | 0049687 + 0025894 + 0128605 + | | AAGP |  | ArielPIN11 |
|  |  | |  |  |  |
| **Laurales** |  | |  |  |  |
| *Persea borbonia* | WIGA-0036428 + ?WIGA-0053901 | | 1KP project |  | PerboPIN1α |
| *Persea borbonia* | WIGA-0026838 + ?WIGA-0053901 | | 1KP project |  | PerboPIN1β |
| *Persea borbonia* | WIGA-0058900 | | 1KP project |  | PerboPIN2α |
| *Persea borbonia* | WIGA-0054389 | | 1KP project |  | PerboPIN2β |
| *Persea borbonia* | WIGA-0028094 | | 1KP project |  | PerboPIN3 |
| *Persea borbonia* | WIGA-0035346 + WIGA-0067030 | | 1KP project |  | PerboPIN5 |
| *Persea borbonia* | WIGA-0059870 | | 1KP project |  | PerboPIN6α |
| *Persea borbonia* | WIGA-0024079 | | 1KP project |  | PerboPIN6β |
| *Persea borbonia* | WIGA-0026372 + ?WIGA-0088839 | | 1KP project |  | PerboPIN11α |
| *Persea borbonia* | WIGA-0039346 + ?WIGA-0088839 | | 1KP project |  | PerboPIN11β |
| *Persea borbonia* | WIGA-0026441 | | 1KP project |  | PerboPIN12 |
|  |  | |  |  |  |
| **Alismatales** |  | |  |  |  |
| *Pistia stratioides* | MFIN-2013271 + MFIN-2051315 | | 1KP project |  | PisstPIN1 |
| *Pistia stratioides* | MFIN-2051430 + MFIN-2009053 | | 1KP project |  | PisstPIN3 |
| *Pistia stratioides* | MFIN-2048977 | | 1KP project |  | PisstPIN5 |
| *Pistia stratioides* | MFIN-2049856 | | 1KP project |  | PisstPIN11 |
| *Posidonia australis* | BYQM-2012160 | | 1KP project |  | PosauPIN1 |
| *Posidonia australis* | BYQM-2009320 | | 1KP project |  | PosauPIN2 |
| *Posidonia australis* | BYQM-2004445 + BYQM-2046685 | | 1KP project |  | PosauPIN3 |
| *Posidonia australis* | BYQM-2062095 | | 1KP project |  | PosauPIN6 |
| *Posidonia australis* | BYQM-2054058 | | 1KP project |  | PosauPIN11 |
| *Triglochin maritimum* | COCP-2006880 | | 1KP project |  | TrimaPIN1 |
| *Triglochin maritimum* | COCP-2007318 | | 1KP project |  | TrimaPIN11 |
|  |  | |  |  |  |
| **Dioscoreales** |  | |  |  |  |
| *Dioscorea villosa* | OCWZ-2007051 | | 1KP project |  | DioviPIN1 |
| *Dioscorea villosa* | OCWZ-2012311 | | 1KP project |  | DioviPIN5 |
| *Dioscorea villosa* | OCWZ-2001225 | | 1KP project |  | DioviPIN6 |
|  |  | |  |  |  |
| **Pandanales** |  | |  |  |  |
| *Freycinetia multiflora* | DGXS-2000812 | | 1KP project |  | FremuPIN1α |
| *Freycinetia multiflora* | DGXS-2098562 | | 1KP project |  | FremuPIN1β |
| *Freycinetia multiflora* | DGXS-2000810 | | 1KP project |  | FremuPIN3α |
| *Freycinetia multiflora* | DGXS-2000809 + DGXS-2104794 | | 1KP project |  | FremuPIN3β |
| *Freycinetia multiflora* | DGXS-2000811 | | 1KP project |  | FremuPIN11 |
| *Freycinetia multiflora* | DGXS-2015776 | | 1KP project |  | FremuPIN12α |
| *Freycinetia multiflora* | DGXS-2002794 | | 1KP project |  | FremuPIN12β |
| *Freycinetia multiflora* | DGXS-2018922 | | 1KP project |  | FremuPIN12γ |
| *Ludovia sp.* | VVVV-2046951 | | 1KP project |  | LudovPIN1 |
| *Ludovia sp.* | VVVV-2010138 | | 1KP project |  | LudovPIN3 |
| *Ludovia sp.* | VVVV-2009719 | | 1KP project |  | LudovPIN5 |
| *Ludovia sp.* | VVVV-2009897 | | 1KP project |  | LudovPIN12α |
| *Ludovia sp.* | VVVV-2009717 | | 1KP project |  | LudovPIN12β |
| *Talbotia elegans* | SILJ-2008241 | | 1KP project |  | TalelPIN1 |
| *Talbotia elegans* | SILJ-2018230 | | 1KP project |  | TalelPIN3α |
| *Talbotia elegans* | SILJ-2017029 | | 1KP project |  | TalelPIN3β |
| *Talbotia elegans* | SILJ-2018837 + SILJ-2080394 | | 1KP project |  | TalelPIN11 |
| *Xerophyta villosa* | QOXT-2008608 | | 1KP project |  | XerviPIN1α |
| *Xerophyta villosa* | QOXT-2018846 + QOXT-2008609 | | 1KP project |  | XerviPIN1β |
| *Xerophyta villosa* | QOXT-2039234 | | 1KP project |  | XerviPIN2 |
| *Xerophyta villosa* | QOXT-2030811 | | 1KP project |  | XerviPIN3 |
| *Xerophyta villosa* | QOXT-2005798 | | 1KP project |  | XerviPIN5 |
| *Xerophyta villosa* | QOXT-2163702 | | 1KP project |  | XerviPIN8 |
|  |  | |  |  |  |
| **Areales** |  | |  |  |  |
| *Sabal bermudana* | HWUP-2023660 | | 1KP project |  | SabbePIN1α |
| *Sabal bermudana* | HWUP-2013526 | | 1KP project |  | SabbePIN1β |
| *Sabal bermudana* | HWUP-2003955 | | 1KP project |  | SabbePIN3α |
| *Sabal bermudana* | HWUP-2003956 | | 1KP project |  | SabbePIN3β |
| *Serenoa repens* | HXJE-2007742 | | 1KP project |  | SerrePIN1 |
|  |  | |  |  |  |
| **Zingiberales** |  | |  |  |  |
| *Canna sp.* | TZNS-2003312 | | 1KP project |  | CannaPIN1e |
| *Canna sp.* | TZNS-2010352 | | 1KP project |  | CannaPIN1f |
| *Canna sp.* | TZNS-2003313 | | 1KP project |  | CannaPIN3 |
| *Curcuma olena* | JQCX-2004453 + JQCX-2006024/OYLU-2003281 | | 1KP project |  | CurolPIN1α |
| *Curcuma olena* | JQCX-2012050/OYLU-2003278 | | 1KP project |  | CurolPIN1β |
| *Curcuma olena* | JQCX-2012051/OYLU-2012713 | | 1KP project |  | CurolPIN1γ |
| *Curcuma olena* | JQCX-2006023/OYLU-2003279 | | 1KP project |  | CurolPIN3 |
| *Heliconia sp.* | TNWF-2013163 | | 1KP project |  | HelicPIN1e |
| *Heliconia sp.* | TNWF-2010992 | | 1KP project |  | HelicPIN1f-α |
| *Heliconia sp.* | KNKV-2009587 | | 1KP project |  | HelicPIN1f-β |
| *Heliconia sp.* | KNKV-2101172 | | 1KP project |  | HelicPIN5 |
| *Orchidantha maxillaroides* | LSKK-2001747 | | 1KP project |  | OrcmaPIN1e |
| *Orchidantha maxillaroides* | LSKK-2079305 | | 1KP project |  | OrcmaPIN6 |
| *Orchidantha maxillaroides* | LSKK-2014553 | | 1KP project |  | OrcmaPIN11 |
| *Strelitzia reginae* | UOEL-2009252 | | 1KP project |  | StrrePIN1e |
| *Strelitzia reginae* | UOEL-2009253 | | 1KP project |  | StrrePIN1f-α |
| *Strelitzia reginae* | UOEL-2009254 | | 1KP project |  | StrrePIN1f-β |
| *Strelitzia reginae* | UOEL-2084221 | | 1KP project |  | StrrePIN3 |
| *Maranta leuconeura* | JNUB-2104602 | | 1KP project |  | MarlePIN6 |
| *Maranta leuconeura* | JNUB-2017636 | | 1KP project |  | MarlePIN11 |
|  |  | |  |  |  |
| **Poales** |  | |  |  |  |
| *Brocchinia reducta* | BYPY-2075055 | | 1KP project |  | BrorePIN1 |
| *Brocchinia reducta* | BYPY-2000209 | | 1KP project |  | BrorePIN3α |
| *Brocchinia reducta* | BYPY-2000211 | | 1KP project |  | BrorePIN3β |
| *Brocchinia reducta* | BYPY-2064941 | | 1KP project |  | BrorePIN5 |
| *Brocchinia reducta* | BYPY-2000212 | | 1KP project |  | BrorePIN11 |
| *Chondropetalum tectorum* | BSTR-2000400 | | 1KP project |  | ChotePIN1α |
| *Chondropetalum tectorum* | BSTR-2000401 | | 1KP project |  | ChotePIN1β |
| *Chondropetalum tectorum* | BSTR-2019115 | | 1KP project |  | ChotePIN11 |
| *Cyperus papyrus* | PWSG-2046223 | | 1KP project |  | CyppaPIN1 |
| *Cyperus papyrus* | PWSG-2007077 | | 1KP project |  | CyppaPIN3 |
| *Juncus inflexus* | CIEA-2051642 + CIEA-2053584 | | 1KP project |  | JuninPIN1 |
| *Joinvillea ascendens* | WXNT-2050509 + WXNT-2054364 | | 1KP project |  | JoiasPIN1 |
| *Joinvillea ascendens* | WXNT-2045133 | | 1KP project |  | JoiasPIN3 |
| *Lepidosperma gibsonii* | WBIB-2014024 | | 1KP project |  | LepgiPIN1 |
| *Lepidosperma gibsonii* | WBIB-2062951 | | 1KP project |  | LepgiPIN2 |
| *Lepidosperma gibsonii* | WBIB-2016923 | | 1KP project |  | LepgiPIN3 |
| *Lepidosperma gibsonii* | WBIB-2006460 | | 1KP project |  | LepgiPIN5α |
| *Lepidosperma gibsonii* | WBIB-2015667 | | 1KP project |  | LepgiPIN5β |
| *Lepidosperma gibsonii* | WBIB-2060377 | | 1KP project |  | LepgiPIN11 |
| *Mapania palustris* | XPAF-2005571 | | 1KP project |  | MappaPIN1 |
| *Mapania palustris* | XPAF-2012295 | | 1KP project |  | MappaPIN3 |
| *Typha angustifolia* | PPQR-2011999 | | 1KP project |  | TypanPIN1 |
| *Typha angustifolia* | PPQR-2001186 | | 1KP project |  | TypanPIN5 |
| *Typha angustifolia* | PPQR-2045199 | | 1KP project |  | TypanPIN11 |
| *Typha latifolia* | BRUD-2002044 | | 1KP project |  | TyplaPIN1α |
| *Typha latifolia* | BRUD-2002767 | | 1KP project |  | TyplaPIN1β |
| *Typha latifolia* | BRUD-2057475 | | 1KP project |  | TyplaPIN6 |
| *Typha latifolia* | BRUD-2012824 | | 1KP project |  | TyplaPIN11 |
| *Oryza sativa* | Os06g12610 | | Phytozome | OsPIN1a | OrysaPIN1a |
| *Oryza sativa* | Os02g50960 | | Phytozome | OsPIN1b | OrysaPIN1b |
| *Oryza sativa* | Os06g44970 | | Phytozome | OsPIN2 | OrysaPIN2 |
| *Oryza sativa* | Os01g45550 | | Phytozome | OsPIN10a | OrysaPIN10a |
| *Oryza sativa* | Os05g50140 | | Phytozome | OsPIN10b | OrysaPIN10b |
| *Oryza sativa* | Os11g04190 | | Phytozome | OsPIN1c | OrysaPIN11α |
| *Oryza sativa* | Os12g04000 | | Phytozome | OsPIN1d | OrysaPIN11β |
| *Oryza sativa* | Os01g69070 | | Phytozome | OsPIN5a | OrysaPIN5a |
| *Oryza sativa* | Os08g41720 | | Phytozome | OsPIN5b | OrysaPIN5b |
| *Oryza sativa* | Os09g32770 | | Phytozome | OsPIN5c | OrysaPIN5c |
| *Oryza sativa* | Os01g51780 | | Phytozome | OsPIN8 | OrysaPIN8 |
| *Oryza sativa* | Os01g58860 | | Phytozome | OsPIN9 | OrysaPIN9 |
| *Sorghum bicolor* | Sb10g008290 | | Phytozome |  | SorbiPIN1a |
| *Sorghum bicolor* | Sb04g028170 | | Phytozome |  | SorbiPIN1b |
| *Sorghum bicolor* | Sb10g026300 | | Phytozome |  | SorbiPIN2 |
| *Sorghum bicolor* | Sb05g002150 | | Phytozome |  | SorbiPIN11 |
| *Sorghum bicolor* | Sb10g004430 | | Phytozome |  | SorbiPIN10b |
| *Sorghum bicolor* | Sb03g043960 | | Phytozome |  | SorbiPIN5a |
| *Sorghum bicolor* | Sb07g026370 | | Phytozome |  | SorbiPIN5b |
| *Sorghum bicolor* | Sb02g029210 | | Phytozome |  | SorbiPIN5c |
| *Sorghum bicolor* | Sb03g032850 | | Phytozome |  | SorbiPIN8 |
| *Sorghum bicolor* | Sb03g037350 | | Phytozome |  | SorbiPIN9 |
| *Zea Mays* | Zm2g074267 | | Phytozome | ZmPIN1b | ZeamaPIN1a |
| *Zea Mays* | Zm2g149184 | | Phytozome | ZmPIN1c | ZeamaPIN1b-α |
| *Zea Mays* | Zm2g098643 | | Phytozome | ZmPIN1a | ZeamaPIN1b-β |
| *Zea Mays* | Zm2g126260 | | Phytozome | ZmPIN10a | ZeamaPIN10a |
| *Zea Mays* | Zm2g160496 | | Phytozome | ZmPIN10b | ZeamaPIN10b |
| *Zea Mays* | Zm2g171702 | | Phytozome | ZmPIN11 | ZeamaPIN11 |
| *Zea Mays* | Zm2g025742 | | Phytozome | ZmPIN5a | ZeamaPIN5a-α |
| *Zea Mays* | Zm2g175983 | | Phytozome |  | ZeamaPIN5a-β |
| *Zea Mays* | Zm2g040911 | | Phytozome | ZmPIN5c | ZeamaPIN5b |
| *Zea Mays* | Zm2g148648 | | Phytozome | ZmPIN5b | ZeamaPIN5c |
| *Zea Mays* | Zm5g839411 | | Phytozome | ZmPIN8 | ZeamaPIN8 |
| *Zea Mays* | Zm5g859099 | | Phytozome | ZmPIN9 | ZeamaPIN9 |
| *Brachypodium distachyon* | Bradi1g45020 | | Phytozome |  | BradiPIN1a |
| *Brachypodium distachyon* | Bradi3g59520 | | Phytozome |  | BradiPIN1b |
| *Brachypodium distachyon* | Bradi1g31530 | | Phytozome |  | BradiPIN2 |
| *Brachypodium distachyon* | Bradi2g44990 | | Phytozome |  | BradiPIN10a |
| *Brachypodium distachyon* | Bradi2g15610 | | Phytozome |  | BradiPIN10b |
| *Brachypodium distachyon* | Bradi4g26300 | | Phytozome |  | BradiPIN11 |
| *Brachypodium distachyon* | Bradi2g58917 | | Phytozome |  | BradiPIN5a |
| *Brachypodium distachyon* | Bradi4g34510 | | Phytozome |  | BradiPIN5b |
| *Brachypodium distachyon* | Bradi3g41080 | | Phytozome |  | BradiPIN5c |
| *Brachypodium distachyon* | Bradi2g48170 | | Phytozome |  | BradiPIN8 |
| *Brachypodium distachyon* | Bradi2g52640 | | Phytozome |  | BradiPIN9 |
|  |  | |  |  |  |
| **Proteales** |  | |  |  |  |
| *Platanus occidentialis* | VQFW-0025202 + ?VQFW-0017798 | | 1KP project |  | PlaocPIN1α |
| *Platanus occidentialis* | VQFW-0025305 + ?VQFW-0017800 | | 1KP project |  | PlaocPIN1β |
| *Platanus occidentialis* | VQFW-0002439 | | 1KP project |  | PlaocPIN3α |
| *Platanus occidentialis* | VQFW-0002111 + VQFW-0001742 | | 1KP project |  | PlaocPIN3β |
| *Platanus occidentialis* | VQFW-0014477 | | 1KP project |  | PlaocPIN6 |
| *Platanus occidentialis* | VQFW-0008690 + VQFW-0000378 | | 1KP project |  | PlaocPIN11α |
| *Platanus occidentialis* | VQFW-0000379 | | 1KP project |  | PlaocPIN11β |
| *Platanus occidentialis* | VQFW-0005588 + VQFW-0008862 | | 1KP project |  | PlaocPIN12 |
| *Hakea drupaceae* | SIIK-0113133 + ?SIIK-0102822 | | 1KP project |  | HakdrPIN1α |
| *Hakea drupaceae* | SIIK-0012194 | | 1KP project |  | HakdrPIN1β |
| *Hakea drupaceae* | SIIK-0113397 | | 1KP project |  | HakdrPIN3 |
| *Hakea drupaceae* | SIIK-0010818 | | 1KP project |  | HakdrPIN5 |
| *Hakea drupaceae* | SIIK-0021763 + SIIK-0035089 | | 1KP project |  | HakdrPIN6 |
| *Hakea drupaceae* | SIIK-0027026 | | 1KP project |  | HakdrPIN12 |
|  |  | |  |  |  |
| **Ranunculales** |  | |  |  |  |
| *Podophyllum peltatum* | WFBF-0042844 + WFBF-0008119 | | 1KP project |  | PodpePIN1α |
| *Podophyllum peltatum* | WFBF-0042951 | | 1KP project |  | PodpePIN1β |
| *Podophyllum peltatum* | WFBF-0018330 | | 1KP project |  | PodpePIN3 |
| *Podophyllum peltatum* | WFBF-0001619 | | 1KP project |  | PodpePIN5 |
| *Podophyllum peltatum* | WFBF-0009526 | | 1KP project |  | PodpePIN11 |
| *Cocculus laurifolius* | LVNW-0026240 | | 1KP project |  | CoclaPIN1 |
| *Cocculus laurifolius* | LVNW-0010317 | | 1KP project |  | CoclaPIN3 |
| *Cocculus laurifolius* | LVNW-0030407 | | 1KP project |  | CoclaPIN5 |
| *Cocculus laurifolius* | LVNW-0116871 | | 1KP project |  | CoclaPIN8 |
| *Cocculus laurifolius* | LVNW-0007909 | | 1KP project |  | CoclaPIN11 |
| *Sanguinaria canadensis* | XHKT-0091709 | | 1KP project |  | SancaPIN1 |
| *Sanguinaria canadensis* | XHKT-0024717 | | 1KP project |  | SancaPIN5 |
| *Sanguinaria canadensis* | XHKT-0013818 | | 1KP project |  | SancaPIN11 |
| *Euptelea pleiosperma* | QTJY-0085988 | | 1KP project |  | EupplPIN1 |
| *Euptelea pleiosperma* | QTJY-0019355 + QTJY-0020824 | | 1KP project |  | EupplPIN3α |
| *Euptelea pleiosperma* | QTJY-0014919 | | 1KP project |  | EupplPIN3β |
| *Euptelea pleiosperma* | QTJY-0019819 | | 1KP project |  | EupplPIN5 |
| *Euptelea pleiosperma* | QTJY-0086451 | | 1KP project |  | EupplPIN6 |
| *Euptelea pleiosperma* | QTJY-0017621 | | 1KP project |  | EupplPIN11 |
| *Euptelea pleiosperma* | QTJY-0085221 + QTJY-0087434 | | 1KP project |  | EupplPIN12 |
| *Papaver rhoeas* | ACYX-0083253 + ACYX-0085588 | | 1KP project |  | PaprhPIN1 |
| *Papaver rhoeas* | ACYX-0027354 | | 1KP project |  | PaprhPIN3 |
| *Papaver rhoeas* | ACYX-0021825 | | 1KP project |  | PaprhPIN5 |
| *Papaver somniferum* | KKCW-0112085 | | 1KP project |  | PapsoPIN1 |
| *Papaver somniferum* | KKCW-0027736 + KKCW-0017158 + KKCW-0027523 | | 1KP project |  | PapsoPIN3 |
| *Papaver somniferum* | MIKW-0028710 | | 1KP project |  | PapsoPIN5 |
| *Papaver somniferum* | MIKW-0045592 | | 1KP project |  | PapsoPIN11 |
| *Aquilegia caerulea* | Aquca_091_00027.1 | | 1KP project |  | AqucaPIN1 |
| *Aquilegia caerulea* | Aquca_018_00005.1 | | Phytozome |  | AqucaPIN2 |
| *Aquilegia caerulea* | Aquca_041_00165.3 | | Phytozome |  | AqucaPIN3 |
| *Aquilegia caerulea* | Aquca_058_00131.1 | | Phytozome |  | AqucaPIN5 |
| *Aquilegia caerulea* | Aquca_074_00070.1 | | Phytozome |  | AqucaPIN8 |
| *Aquilegia caerulea* | Aquca_009_00440.1 | | Phytozome |  | AqucaPIN11 |
|  |  | |  |  |  |
| **Gunnerales** |  | |  |  |  |
| *Gunnera manicata* | XMQO-0165671 | | 1KP project |  | GunmaPIN1 |
| *Gunnera manicata* | XMQO-0161399 + XMQO-0160791 | | 1KP project |  | GunmaPIN3 |
|  |  | |  |  |  |
| **Trochodendrales** |  | |  |  |  |
| *Trochodendron araliodes* | SWOH-0025439 | | 1KP project |  | TroarPIN1α |
| *Trochodendron araliodes* | SWOH-0098732 + SWOH-0095885 | | 1KP project |  | TroarPIN1β |
| *Trochodendron araliodes* | SWOH-0096653 + SWOH-0022253 | | 1KP project |  | TroarPIN3α |
| *Trochodendron araliodes* | SWOH-0014411 + SWOH-0023699 + ?SWOH-0090639 | | 1KP project |  | TroarPIN3β |
| *Trochodendron araliodes* | SWOH-0094534 + ?SWOH-0017388 | | 1KP project |  | TroarPIN3γ |
| *Trochodendron araliodes* | SWOH-0017524 | | 1KP project |  | TroarPIN11 |
| *Trochodendron araliodes* | SWOH-0097094 | | 1KP project |  | TroarPIN12 |
|  |  | |  |  |  |
| **Berberidopsiales** |  | |  |  |  |
| *Aextoxicon punctatum* | QUTB-0112274 | | 1KP project |  | AexpuPIN1 |
| *Aextoxicon punctatum* | QUTB-0112302 | | 1KP project |  | AexpuPIN3 |
| *Aextoxicon punctatum* | QUTB-0104941 | | 1KP project |  | AexpuPIN5 |
| *Aextoxicon punctatum* | QUTB-0022232 | | 1KP project |  | AexpuPIN6 |
| *Aextoxicon punctatum* | QUTB-0026342 | | 1KP project |  | AexpuPIN11 |
|  |  | |  |  |  |
| **Brassicales** |  | |  |  |  |
| *Arabidopsis thaliana* | At1g73590 | | TAIR | AtPIN1 | ArathPIN1 |
| *Arabidopsis thaliana* | At5g57090 | | TAIR | AtPIN2 | ArathPIN2 |
| *Arabidopsis thaliana* | At1g70940 | | TAIR | AtPIN3 | ArathPIN3 |
| *Arabidopsis thaliana* | At2g01420 | | TAIR | AtPIN4 | ArathPIN4 |
| *Arabidopsis thaliana* | At1g23080 | | TAIR | AtPIN7 | ArathPIN7 |
| *Arabidopsis thaliana* | At5g16530 | | TAIR | AtPIN5 | ArathPIN5 |
| *Arabidopsis thaliana* | At1g77110 | | TAIR | AtPIN6 | ArathPIN6 |
| *Arabidopsis thaliana* | At5g15100 | | TAIR | AtPIN8 | ArathPIN8 |
| *Arabidopsis lyrata* | 476492 | | Phytozome |  | AralyPIN1 |
| *Arabidopsis lyrata* | 908693 | | Phytozome |  | AralyPIN3 |
| *Arabidopsis lyrata* | 484057 | | Phytozome |  | AralyPIN4 |
| *Arabidopsis lyrata* | 472559 | | Phytozome |  | AralyPIN7 |
| *Arabidopsis lyrata* | 950383 | | Phytozome |  | AralyPIN2 |
| *Arabidopsis lyrata* | 927110 | | Phytozome |  | AralyPIN6 |
| *Arabidopsis lyrata* | 326097 | | Phytozome |  | AralyPIN5 |
| *Arabidopsis lyrata* | 325967 | | Phytozome |  | AralyPIN8 |
| *Carica papaya* | evm.TU.supercontig_127.48 | | Phytozome |  | CarpaPIN12 |
|  |  | |  |  |  |
| **Malpighiales** |  | |  |  |  |
| *Populus trichocarpus* | Pt0012s04470 | | Phytozome | PtrPIN7 | PoptrPIN1α |
| *Populus trichocarpus* | Pt0015s04570 | | Phytozome | PtrPIN1 | PoptrPIN1β |
| *Populus trichocarpus* | Pt0018s13610 | | Phytozome | PtrPIN9 | PoptrPIN2α |
| *Populus trichocarpus* | Pt0001s21230 | | Phytozome | PtrPIN10 | PoptrPIN2β |
| *Populus trichocarpus* | Pt0010s12320 | | Phytozome | PtrPIN3 | PoptrPIN3α |
| *Populus trichocarpus* | Pt0008s12830 | | Phytozome | PtrPIN6 | PoptrPIN3β |
| *Populus trichocarpus* | Pt0006s03540 | | Phytozome | PtrPIN8 | PoptrPIN11α |
| *Populus trichocarpus* | Pt0016s03450 | | Phytozome | PtrPIN2 | PoptrPIN11β |
| *Populus trichocarpus* | Pt0019s07990 | | Phytozome | PtrPIN12 | PoptrPIN5α |
| *Populus trichocarpus* | Pt0013s08510 | | Phytozome | PtrPIN11 | PoptrPIN5β |
| *Populus trichocarpus* | Pt0005s20990 | | Phytozome | PtrPIN4 | PoptrPIN6α |
| *Populus trichocarpus* | Pt0002s07310 & Pt0002s07300 | | Phytozome | PtrPIN5 | PoptrPIN6β |
| *Populus trichocarpus* | Pt0017s11440 | | Phytozome | PtrPIN14 | PoptrPIN8α |
| *Populus trichocarpus* | Pt0004s12310 | | Phytozome | PtrPIN13 | PoptrPIN8β |
| *Populus trichocarpus* | Pt0014s14390 | | Phytozome | PtrPIN15 | PoptrPIN12 |
|  |  | |  |  |  |
| **Vitales** |  | |  |  |  |
| *Vitis vinifera* | GSVIVT00017824001 | | Phytozome |  | VitviPIN1 |
| *Vitis vinifera* | GSVIVT00031315001 | | Phytozome |  | VitviPIN2 |
| *Vitis vinifera* | GSVIVT00030482001 | | Phytozome |  | VitviPIN3 |
| *Vitis vinifera* | GSVIVT00023254001 | | Phytozome |  | VitviPIN11α |
| *Vitis vinifera* | GSVIVT00023255001 | | Phytozome |  | VitviPIN11β |
| *Vitis vinifera* | GSVIVT00025108001 | | Phytozome |  | VitviPIN5α |
| *Vitis vinifera* | GSVIVT01019110001 | | Phytozome |  | VitviPIN5β |
| *Vitis vinifera* | GSVIVT00014302001 | | Phytozome |  | VitviPIN6 |
| *Vitis vinifera* | GSVIVT00020886001 | | Phytozome |  | VitviPIN8 |
| *Vitis vinifera* | GSVIVT00033553001 | | Phytozome |  | VitviPIN12α |
| *Vitis vinifera* | GSVIVT01031663001 | | Phytozome |  | VitviPIN12β |
|  |  | |  |  |  |
| **Solanales** |  | |  |  |  |
| *Solanum lycopersicon* | Solyc03g118740 | | Solgenomics | SlPIN1 | SollyPIN1 |
| *Solanum lycopersicon* | Solyc07g006900 | | Solgenomics | SlPIN2 | SollyPIN2 |
| *Solanum lycopersicon* | Solyc05g008060 | | Solgenomics | SlPIN4 | SollyPIN3α |
| *Solanum lycopersicon* | Solyc04g007690 | | Solgenomics | SlPIN3 | SollyPIN3β |
| *Solanum lycopersicon* | Solyc10g078370 | | Solgenomics | SlPIN9 | SollyPIN11α |
| *Solanum lycopersicon* | Solyc10g080880 | | Solgenomics | SlPIN7 | SollyPIN11β |
| *Solanum lycopersicon* | Solyc01g068410 | | Solgenomics | SlPIN5 | SollyPIN5α |
| *Solanum lycopersicon* | Solyc04g056620 | | Solgenomics | SlPIN10 | SollyPIN5β |
| *Solanum lycopersicon* | Solyc06g059730 | | Solgenomics | SlPIN6 | SollyPIN6 |
| *Solanum lycopersicon* | Solyc02g087660 | | Solgenomics | SlPIN8 | SollyPIN8 |
|  |  | |  |  |  |
| **Lamiales** |  | |  |  |  |
| *Mimulus Guttatus* | mgv1a003175m | | Phytozome |  | MimguPIN1α |
| *Mimulus Guttatus* | mgv1a003655m | | Phytozome |  | MimguPIN1β |
| *Mimulus Guttatus* | mgv1a003446m | | Phytozome |  | MimguPIN11 |
| *Mimulus Guttatus* | mgv1a002731m | | Phytozome |  | MimguPIN3α |
| *Mimulus Guttatus* | mgv1a025638m | | Phytozome |  | MimguPIN3β |
| *Mimulus Guttatus* | mgv1a002534m | | Phytozome |  | MimguPIN2 |
| *Mimulus Guttatus* | mgv1a004829m | | Phytozome |  | MimguPIN6 |
| *Mimulus Guttatus* | mgv1a024604m | | Phytozome |  | MimguPIN8 |
| *Mimulus Guttatus* | mgv1a007953m | | Phytozome |  | MimguPIN5α |
| *Mimulus Guttatus* | mgv1a009388m | | Phytozome |  | MimguPIN5β |
